# Safety of a co-designed cognitive behavioural therapy intervention for people with type 1 diabetes and eating disorders (STEADY): a feasibility randomised controlled trial

**DOI:** 10.1016/j.lanepe.2024.101205

**Published:** 2025-01-20

**Authors:** Marietta Stadler, Natalie Zaremba, Amy Harrison, Jennie Brown, Divina Pillay, Jacqueline Allan, Rachael Tan, Salma Ayis, Emmanouela Konstantara, Janet Treasure, David Hopkins, Khalida Ismail

**Affiliations:** a Department of Diabetes, Faculty of Life Sciences and Medicine, King's College London, London, UK; b Department of Psychological Medicine, Diabetes, Psychology and Psychiatry Research Group, King's College London, London, UK; c Department of Medical Psychology, Radboud University Medical Center, Nijmegen, the Netherlands; d Department of Psychology and Human Development, University College London, Institute of Psychiatry, London, UK; e Department of Diabetes, King's College Hospital, London, UK; f School of Population Health& Environmental Sciences, King's College London, UK; g Institute of Psychiatry, Psychology and Neuroscience, King's College London, London, UK; h Diabetes Endocrinology and Obesity, King's Health Partners, London, UK

**Keywords:** Type 1 diabetes, Eating disorder, Cognitive behavioural therapy, Experience based co-design, Randomised controlled trial

## Abstract

**Background:**

Safe management of people with Type 1 diabetes and EAting Disorders studY (STEADY) is a complex intervention for people with type 1 diabetes and mild-to-moderate disordered eating (T1DE) integrating cognitive behavioural therapy (CBT) with diabetes education. Aim was to test feasibility of STEADY in a randomised controlled trial.

**Methods:**

Feasibility parallel-group, randomised (blocks of four) controlled open-label trial (RCT) of STEADY against usual care (Control) at King's College London, UK. Participants were referred by clinicians or self-referred via social media advertisements. Forty adults with T1DE (Hba1c < 15%, body mass index 15–35 kg/m^2^, age ≥ 18 years) were randomised. STEADY was delivered in 12 sessions by a CBT-trained Diabetes Specialist Nurse through video-conferencing and mobile app. Main outcome at 6 months post-randomisation was feasibility. Baseline mental health data (Structured Clinical Interview for DSM-5, SCID-5RV), and secondary biomedical outcomes (HbA1c; glucose time in range; TIR) and person-reported outcome measures (PROM: Diabetes Eating Problems Survey-Revised, DEPS-R; Eating Disorder Examination Questionnaire Short, EDE-QS; Type 1 Diabetes Distress Scale, T1DDS; Generalised Anxiety Disorder Assessment, GAD-7; Patient Health Questionnaire, PHQ-9; Impact of Diabetes Profile, DIDP) were collected. Analyses were conducted as intention-to-treat. ClinicalTrials.govNCT05140564.

**Findings:**

Of the 98 screened, 40 participants with T1DE were randomised (recruitment rate: 40.81%; 95% CI: 31.60%, 50.72%): 38 women, 1 man, 1 trans man (37 White, 1 White/Asian, 1 Black; 39 ± 11 years old, diabetes duration 22 ± 15 years, HbA1c 9.1 ± 2.6%). The drop-out rate was 3/20 = 15% (4.39%, 36.55%) in STEADY and 2/20 = 10% (1.57%, 31.32%) in Control. STEADY reported lower GAD-7 (5.75 ± 2.89 vs 10.18 ± 5.31, p = 0.0060) and higher DIDP (3.13 ± 0.63 vs 2.46 ± 0.87, p = 0.020) at follow-up compared with Control, indicating lower anxiety and higher diabetes-specific quality-of-life. Compared to baseline, STEADY improved in DEPS-R, EDE-QS, GAD-7, PHQ-9 and T1DDS.

**Interpretation:**

The STEADY-feasibility RCT demonstrated proof-of-concept for feasibility and mental health improvements in T1DE without deteriorating glycaemic control. A full scale RCT of STEADY will test effectiveness and implementation.

**Funding:**

10.13039/501100000272National Institute for Health Research (CS-2017-17-023).

**Research in context**

**Evidence before this study**

Type 1 diabetes with comorbid disordered eating (T1DE) is a difficult to treat condition with increased morbidity and mortality rates. Our systematic review and meta-analysis searched Medline, Embase, PsycINFO, the Cochrane Library, PubMed and OpenGrey databases up to 8/2016 to identify randomised and non-randomised controlled trials in people with T1DE (4), using the main search terms ‘type 1 diabetes’, ‘psychotherapeutic’ or ‘psychoeducational interventions’, and ‘disordered eating’. Six out of 91 abstracts reviewed met the inclusion criteria. There was insufficient data to pool for disordered eating outcomes, meta-analyses for glycaemic control could be conducted from 3 with a pooled sample size n = 118), only one was a randomised controlled trial (RCT). There was no statistically significant improvement in glycaemic control the treatment group compared with comparison group, inpatient therapy appeared to be the most effective treatment. Since this review, we have reviewed the literature once more for a systematic review on the definition of T1DE in research projects as part of a PhD project (last update 7/2023), which identified only 1 feasibility trial testing self-compassion in n = 27 adolescents with T1D and moderate disordered eating behaviours which did not report on clinical outcomes and one uncontrolled feasibility pilot trial testing an intervention grounded in acceptance and commitment therapy, showing improvements in eating disorders symptoms and diabetes distress. There is no effective intervention that integrates type 1 diabetes and eating disorders treatment for those with mild to moderate presentations of T1DE in the outpatient setting.

**Added value of this study**

This is the first intervention that has been co-designed with people with lived experience of T1DE and that used the experience-based-co-design methodology to inform the development of the complex intervention. This is the first T1DE intervention that fully integrated all aspects of diabetes related mental health with diabetes physical health, responding to the needs of the majority of T1DE patients who have mental health diagnoses additional to their eating disorder. The Safe management of people with Type 1 diabetes and EAting Disorders studY (STEADY) intervention is a diabetes specific cognitive behavioural therapy (CBT) intervention delivered by a trained and competent diabetes health care professional. It is innovative in that it can be delivered in person or fully virtually, whilst the patient remains embedded in their usual care setting, ensuring continuity of health care delivery. This feasibility RCT demonstrated that STEADY recruitment is feasible and the drop-out rates are low. Mental health outcomes improved whilst glycaemic control did not deteriorate.

**Implications of all the available evidence**

It is feasible to conduct an RCT of the integrated diabetes and mental health intervention STEADY for people with mild to moderate disordered eating in type 1 diabetes. There is proof of concept of improvement of psychological outcome measures. It will be feasible to test STEADY's effectiveness and implementation in a multi-centre RCT in view of implementing it into routine diabetes care.

## Introduction

Type 1 diabetes (T1D) and disordered eating (T1DE) is a complex and dangerous comorbidity that is difficult to treat and associated with high morbidity and mortality.^1^ T1DE presents with difficulties in diabetes self-care behaviours, which can include deliberate insulin omission or restriction, low-carbohydrate diets to reduce insulin requirements or blood glucose fluctuations, and episodes of binge eating in response to hypoglycaemia symptoms.^2^^,^^3^ Currently, no intervention effectively addresses both the T1D targets and mental health outcomes in T1DE,^4^ particularly for people with mild to moderate presentations. Our group recently evaluated a service for those with severe T1DE that delivers high-intensity multidisciplinary care, including inpatient treatment for a small number of patients.^5^ The Safe management of people with Type 1 diabetes and EAting Disorders studY (STEADY) intervention was developed to offer an outpatient CBT brief focused-intervention for those with less severe T1DE which can be delivered by a trained diabetes health care professional.

We developed STEADY using the Experience-Based Co-Design (EBCD) process,^6^ following the Medical Research Council guidelines for developing complex interventions.^7^ It was informed by our theoretical model of T1DE, developed from literature reviews,^8^ a thematic analysis of blogs written by people with T1DE,^3^ semi-structured interviews with people with T1DE,^2^^,^^9^ and focus groups with healthcare professionals.^10^ STEADY is a novel, complex, CBT-based intervention that has been co-designed by people with lived experience of T1DE, and healthcare professionals experienced in treating T1DE.^6^^,^^11^

This study aims to test the STEADY intervention in a feasibility randomised controlled trial (RCT) of 12 sessions of STEADY-CBT compared with usual care in people with mild to moderate T1DE. The primary outcome feasibility parameters were recruitment and attrition rates and deriving a standard deviation of glycated haemoglobin A1c (HbA1c) and sensor glucose time in range (TIR) to inform a sample size calculation for a definitive RCT. The secondary outcomes of biomedical measures were HbA1c, TIR, acute diabetes complication rates and of psychometric measures were person reported outcome measures.

## Methods

### Study design

This was a double-arm, parallel-group, open-label, randomised feasibility trial comparing the STEADY intervention (STEADY group) with usual care (Control group).

The trial was set in King's College Hospital, London UK, with baseline and end-of-study appointments conducted at the King's College Hospital Clinical Trials Facility (https://ctu.co.uk/).

This trial complied with the relevant regulations for the conduct of research in human volunteers (in accordance with the Declaration of Helsinki in its latest form). Ethical approval was obtained from the East of England–Essex Research Ethics Committee (21/EE/0235). One non-substantial amendment (NSA01, dated 13/12/2022) reflected a cost extension (owing to the Covid-19 pandemic) and updated the study end date to 28/02/2024.

One substantial amendment (SA01, dated 24/4/2023), following an SAE not related to the study, included updates to our protocol and participant information sheet to further detail the procedures when the trial team would contact the participant's usual care teams to assess safety and eligibility in the trial. We also further clarified the purpose of the diabetes education and mental health crisis plan, for participants to reference and signpost to their usual care teams or A&E if applicable, in case of emergency.

Deviations from the protocol monitored and recorded in real-time were minor: Timelines from randomisation to follow up were delayed by 3 months–9 months, but when measured from the first therapy session to the end of study appointment, this reduced to a mean of 6.5 months: Our study therapist had to delay the start of therapy with newly randomised participants until participants in her active caseload were able to finish therapy; some participants requested longer times between therapy sessions. One participant was included in the study based on an Hba1c at their screening appointment below the 15% exclusion cut-off and was randomised before the baseline laboratory result of the HbA1c was reviewed (which was above cut-off). The participant remained in the study as they met all other inclusion criteria.

Trial progress and safety were monitored by a trial steering committee. The protocol is available on ClinicalTrials.gov (trial registration number NCT05140564) and has been published as a peer-reviewed paper.^11^

### Participants

Adults (18 years or older) with established T1D (≥6 months) experiencing disordered eating or an eating disorder (T1DE) were eligible. T1DE was defined as restriction or manipulation of insulin to control weight, food restriction, binge eating, or any additional disordered eating behaviour as described by the Diagnostic and Statistical Manual of Mental Disorders (DSM-5 (12)), or International Classification of Diseases 11th Revision (ICD-11), or a score of ≥15 on the Eating Disorder Examination Questionnaire Short (EDE-QS),^12^ or a score of ≥20 on the Diabetes Eating Problem Survey- Revised (DEPS-R^13^).

T1DE was confirmed at the baseline visit using: i) a Structured Clinical Interview using DSM-5 (SCID-5RV)^14^ ii) additional T1DE-specific behavioural criteria developed by the study team, following the Experience-Based Co-Design work^2^^,^^6^) iii) the DEPS-R and EDE-QS questionnaires. Additional inclusion criteria were willingness to participate in the STEADY intervention (12 sessions of T1DE-specific CBT and diabetes education) or the control group, being currently under the care of a diabetes specialist team, ability to speak, write, and read in English, and capacity and able to provide written informed consent.

Key exclusion criteria were body mass index (BMI) below 15 kg/m^2^, HbA1c > 15% (140.4 mmol/mol), pregnancy or planning pregnancy, recurrent acute diabetes complications (>2 episodes of severe hypoglycaemia requiring third-party assistance; >2 admissions for diabetic ketoacidosis in the past 12 months), severe mental illness requiring acute and urgent treatment (severe depression with suicidal ideation, psychosis, emotionally unstable personality disorder requiring more intensive psychiatric treatment substance problem use and dependence), advanced diabetes complications (end-stage renal disease, limb amputation, registered blind), uncontrollable electrolyte disturbance, low blood pressure (<100/60 mmHg), or other physical conditions requiring inpatient treatment. These exclusion criteria were chosen to ensure the safety of participants in an outpatient-based trial and suitability to be randomised into the control group. Potential participants that consented and were excluded after the baseline visit, because they were not eligible, were referred to their primary care and local services.

Eligible people expressing interest in response to advertisements on social media or study information provided by their clinician contacted the study team via email. They were invited to a telephone screening visit and, if eligible and willing, provided written consent and were offered a face-to-face baseline visit.

A joint multidisciplinary baseline clinical assessment was conducted by a study diabetologist, psychiatrist and clinical psychologist to establish whether the participant was safe to proceed with STEADY or required referral/signposting to a different clinical treatment or therapy.

### Randomisation and masking

Following baseline eligibility assessment, 40 participants were randomly assigned (1:1) to either the STEADY intervention or the control group using 4 by 4 block sizes. The randomisation was performed using a centralised software system provided by the King's Clinical Trials Unit. The outcome of the randomisation was sent digitally to the trial manager and communicated to each participant by the study team. Participants and the study team were not masked to group allocation. The trial statistician supervising the data analysis remained blinded to the group allocation.

### Procedures

Participants in the STEADY group received up to 12 sessions of T1DE-specific CBT delivered by a diabetes specialist nurse (DSN). The participant's routine diabetes care (and eating disorder care) remained with their usual care teams.

Participants in the control group continued to receive usual medical care from their local diabetes team and their usual care team were recommended to make a referral to local eating disorder services as recommended by NICE.

The STEADY intervention integrates diabetes education, diabetes- CBT and T1DE-specific modules that were co-designed with people with lived experience of T1DE and health care professionals in a multidisciplinary iterative process. The detailed description of the experience-based co-design (EBCD) process has been published.^6^ STEADY uses a modular approach tailored to each participant's needs,^11^ depending on the initial psychotherapeutic formulation. A key component of STEADY is behavioural experiments involving gradual changes to diabetes management behaviours e.g., titration of insulin injection at a pace that is medically safe and that is tolerable to the participant. Other components of the toolkit address binge eating, food restriction, fear of hypoglycaemia, exercise adjustments, perfectionism and acceptance of diabetes.^2^^,^^6^

The table of contents of the STEADY therapy manual is shown in Supplemental Table S1a (Appendix, page 1–2).

The DSN delivering the intervention (JB) has extensive experience in managing T1D and had a Post Graduate Diploma in CBT (equivalent to the NHS definition of high intensity CBT). The clinical psychologist (AH) has extensive experience in eating disorders, trained in CBT and a CBT trainer, and provided weekly supervision to the DSN lasting 90 min where each active participant was discussed. The DSN and the psychologist were part of the multidisciplinary team that developed the STEADY toolkit and ensured all materials in the STEADY toolkit were appropriate in the context of both CBT and diabetes education.^11^ The study clinical psychologist (AH) was trained in the basics of T1D and provided STEADY therapy to participants with low medical risk (no admissions in diabetic ketoacidosis, DKA or severe hypoglycaemia, SH) under the supervision of the study DSN and diabetologist to provide additional capacity for participants to begin the intervention. The psychiatrist (DP), and study physician (MS) provided supervision and clinical input for mental health and medical issues respectively.

At the baseline visit, comprehensive physical health, diabetes, and mental health histories were taken, a blood sample for HbA1c measurement was collected. Demographic data and questionnaires for person-reported outcome measures (PROM)^15^, ^16^, ^17^, ^18^, ^19^, ^20^ were collected electronically via Redcap®. Demographic data, including sex assigned at birth and gender identity, were self-reported. Diabetes therapy, including insulin pump settings, and mental health therapy were reported to the study professional conducting the baseline visit, along with self-reported rates of hospital admissions related and un-related to diabetes, episodes of severe hypoglycaemia in the past 12 months. Blood pressure, body weight and height were measured. HbA1c was measured at King's College Hospital (ViaPath) using High Pressure Liquid Chromatography (Premier 9210 analyser, Menarini, Italy). Continuous or flash glucose monitoring (CGM/fCGM) data of devices worn for routine clinical care were downloaded for further analysis. Routinely obtained blood reports were requested from the participant's general practitioner (GP) or diabetes care provider.

At the baseline visit, basic aspects of diabetes education were covered in a one-to-one session with the diabetologist using standard diabetes educational material adjusted to disordered eating to ensure that all participants had sufficient basic education on the treatment of hypoglycaemia, sick day rules, and safe injection techniques. The study psychiatrist or clinical psychologist conducted a detailed mental health assessment, eating disorder history and the Structured Clinical Interview for DSM-5,^14^ which entailed detailed classification and documentation of mental health diagnoses, ongoing and past history; An individualised ‘mental health crisis plan’ was developed with the participant to ensure adequate signposting in the event it was needed. A brief physical examination was conducted when required (e.g., assessing lipodystrophy, checking feet).

The STEADY intervention was delivered one-on-one over up to 12 sessions. The 1 h long STEADY therapy visits were conducted virtually over Zoom (https://zoom.us/) which was chosen for the whiteboard feature helping facilitate CBT exercises in-session. Virtual therapy sessions provided flexibility and safety to research participants and staff during the COVID-19 pandemic. Participants had the option to use a bespoke STEADY smartphone application to facilitate therapy delivery and communication between the participant and DSN, or they could receive all materials in a paper format or via email. The STEADY app and digital health platform were hosted by Living With® (www.livingwith.health).

In the first therapy session, participants were introduced to the fundamental components of STEADY-specific CBT, and the therapist worked with the participant to develop a CBT formulation. Participants also set themselves three individualised biopsychosocial recovery goals, which include mental health, physical health, and social goals, and rated their progress on a 0–10 scale throughout the course of their therapy sessions.

Sessions 2 to 11 followed a structured format where the therapist and participant worked together towards goals they set: 1) Brief check-in containing standard items (mood, blood glucose levels, ketone levels, insulin), 2) Summary of the previous session, 3) Agenda setting to agree on the focus of the session, 4) Review of the agreed independent therapy exercises completed since the last session (“active practice”). Main agenda items usually included: 1) Re-setting or reviewing progress towards previously agreed goals, 2) Setting new “active practice” tasks informed by the session, 3) A final summary of what has been learned during the session.

The first four therapy sessions were conducted weekly to fortnightly, and the final eight sessions were scheduled at times which suited the participant's needs (weekly to monthly intervals). Participants recorded their CBT active practice and notes in the STEADY app or paper worksheets between sessions. Before each session, participants were asked to complete the DEPS-R,^13^ PHQ-9,^21^ and GAD-7.^22^ The study DSN sent participants their summary of the session on the STEADY app or via email. Completion of 6 or more sessions was considered as intervention completion, attending the follow up appointment as study completion. The final STEADY session summarised the participant's progress and reviewed relapse prevention.

The end of study visit occurred 6 months post-randomisation (±6 weeks) in-person for all participants, with a review of mental and physical health and diabetes care with repeated data collection and recording of adverse events and changes in therapy (medication, therapy modality, diabetes technology).

A remote extended observation visit will occur 12 months post-randomisation via telephone or videoconference as data collection is ongoing including routine blood reports, baseline questionnaires, and hospital admissions. Both intervention and control groups will participate in this extended observation.

### Outcomes

The primary outcome was the feasibility of delivering the STEADY intervention, including recruitment and attrition rates, and deriving a standard deviation of HbA1c and sensor glucose time in range (TIR) to inform a sample size calculation for a definitive trial.

Secondary outcomes included biomedical measures (HbA1c, TIR, acute diabetes complication rates), and psychometric measures in the form of PROM (Supplemental Table S2, Appendix, page 4).

Safety and adverse event data were collected during the follow-up visit for both groups and in real-time, when the trial team was made aware of them. Participants could contact the study team via a STEADY trial email address, monitored during working hours. Primary care practitioners had received a letter with study participation information and study team contact details. We advised participants that STEADY was not a crisis service and to seek emergency care from their diabetes team or attend accident and emergency facilities as the nearest hospital if needed. Data were collected and reported for serious adverse events, defined as any untoward medical occurrence resulting in death, life-threatening events, unplanned inpatient hospital admissions, or resulting in persistent or significant disability/incapacity.

Safety outcomes data were collected and presented in Supplemental Table S4 (Appendix, page 6). Severe hypoglycaemic episodes (requiring third-party assistance), and DKA (defined as hospital admission with confirmed DKA, pH ≤ 7.30, bicarbonate ≤18 mmol/L, and serum ketones> 3 mmoL/l) were recorded but not classified as severe adverse events due to their common presentation in T1DE.

### Statistical analyses

A sample size of 40 participants provided sufficient precision for estimating a pooled standard deviation (SD) for HbA1c, for subsequent use in a sample size calculation of a definitive trial. Assuming a SD of 2% (based on the eligible range of HbA1c of up to 15%), this gives a 95% CI of 1.64%–2.57%. Assuming an attrition rate of 10%, our sample size gives a 95% CI of 3%–24%. This provides sufficient precision for the calculation of rates such as recruitment rates (calculations were performed using the PASS® v15 software).^11^

Recruitment and dropout rates were calculated and presented with 95% CI. The pooled SD of HbA1c and TIR at baseline and end of study were also calculated. Descriptive statistics were calculated for the STEADY and control groups at baseline. Intention-to-treat (including all randomised participants) analysis, as well as per-protocol analysis (including all participants who completed the study) were conducted, comparing the difference in outcomes at follow up against the baseline and between groups. The change in HbA1c and TIR was calculated for both intervention and control groups, and descriptive statistics are presented to provide information about proof-of-concept for the effectiveness of STEADY. PROM data were assessed for normality using the Shapiro–Wilk test. For consistency, questionnaires with multiple subscales were treated as normally distributed if the total score or ≥50% of subscales were normally distributed. Paired t-tests were conducted to compare baseline and study end PROM scores for normally distributed PROM, effect sizes were calculated using Cohen's d. Non-parametric Man-Whitney U test was conducted for non-normally distributed EDE-QS scores.

All analyses were completed in SPSS version 29.0.2.0.^23^ We created an electronic case report form (eCRF) using REDCap, which is General Data Protection Regulation (GDPR) and Health Insurance Portability and Accountability Act (HIPPA) compliant.^24^ Data was anonymised and stored using a unique participant identifier. Data was entered into REDCap directly, including questionnaires and relevant source data such as continuous glucose monitors (CGM) and glucose meter downloads.

### Role of the funding source

This work was conducted as part of the National Institute for Health and Care Research (NIHR) funded STEADY project (Safe management of people with Type 1 diabetes and EAting Disorders studY; CS-2017- 17–023) through the Clinician Scientist Fellowship awarded to MS. The funder of the study (NIHR) had no role in study design, data collection, data analysis, data interpretation, or writing of the report.

## Results

**Fig. 1:**
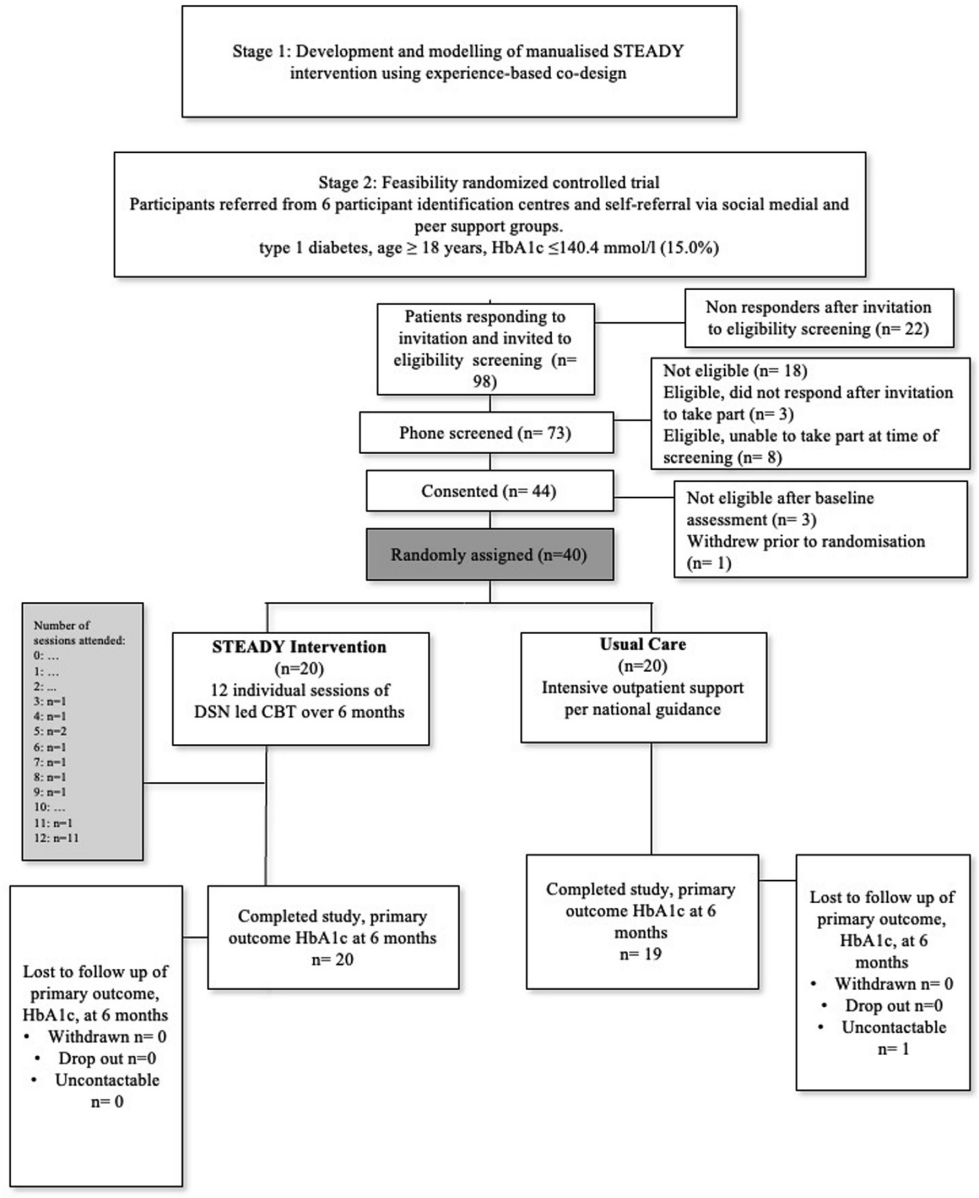
Study flow chart of feasibility randomised controlled trial comparing STEADY with usual care in people with type 1 diabetes and eating disorders.

Between 14.02.2022 and 22.08.2023, 98 people expressed interest in participating; 73 attended a phone screening for eligibility; 18 (25%) were not eligible and 11 (15%) were not able or willing to attend the baseline visit. We assessed 44 (60%) participants at baseline, and 40 eligible participants were randomly assigned (between 24.05.2022 and 29.09.2022), with randomisation taking place 6.2 weeks (IQR: 1.4–10) after baseline assessment. Three participants were excluded at their baseline visit, before randomisation, due to exclusion criteria related to the mental health or physical health assessment (recent mental health crisis with suicidality; recurrent severe hypoglycaemia; very severe and unstable eating disorder with significant mental health comorbidity and suicidality), and one withdrew before randomisation. Twenty participants were randomised into STEADY and 20 into the Control groups (Fig. 1).

Seventeen participants in the STEADY arm completed the study, of which 16 completed both the therapy and the final study visit, one of the 16 completed the follow up remotely via videocall, and one participant completed therapy but did not attend the final study visit and their HbA1c and TIR data collected remotely. Eighteen participants in the control group completed the study and attended the final study visit, two of which attended the follow up visit remotely via videocall. HbA1c and hospital admission data from participants who had withdrawn from the study or were not contactable at follow up was collected from their GPs with their consent (n = 4). The total time in the study from randomisation was 32 weeks (IQR: 30–46). The use of modular worksheets within the STEADY manual (“toolkit”) is shown in Supplemental Table S1a (Appendix, pages 1–2), covering a range of generic CBT tools (e.g., formulation used for each participant), those targeting diabetes-related thoughts and behaviours (e.g., perfectionism), and those targeting specific T1DE behaviours (e.g., binge eating, insulin restriction) Supplemental Table S1b (Appendix, page 3).

Baseline characteristics were similar between groups (Table 1). Participants (38 women, 1 man, 1 trans man) were 39.4 ± 11.2 years old, had a diabetes duration of 22.0 ± 14.7 years. The pooled baseline HbA1c was 9.1% ± 2.6% (75.6 ± 28.6 mmol/mol) and the pooled TIR at baseline 46.3 ± 30.0%. All participants had DSM-5 diagnosable eating disorders (Other Specified Feeding or Eating Disorder, OSFED n = 24; Bulimia Nervosa, BN, n = 14; Binge Eating Disorder, BEN, n = 2; Table 1), with 29 (72.5%) having one or more additional psychiatric DSM-5 diagnoses (15 had one additional DSM-5 diagnosis, 10 had 2 additional DSM-5 diagnoses and 4 had 4 or more DSM-5 diagnoses) (Table 1). Most participants (57.5%) were using multiple daily injection therapy, and all but one were routinely using continuous glucose monitoring systems at baseline.

**Table 1:** Baseline characteristics of the intention-to-treat population of the STEADY trial.

	N/N	STEADY (n = 20)	Control (n = 20)
**Anthropometric data**			
Sex at birth	20/20		
Female		19 (95%)	20 (100%)
Male		1 (5%)	0 (0%)
Gender	20/20		
Women		18 (90%)	20 (100%)
Men		2 (10%)	0 (0%)
Age (years)	20/20	35.5 (30.5–48.8)	34.4 (31.7–47.7)
Ethnic origin	19/20		
White–English/Welsh/Scottish/Northern Irish/British		17 (85%)	17 (85%)
White – Gypsy or Irish Traveller		1 (5%)	0 (0%)
White- any other background		0 (0%)	2 (10%)
Black–Caribbean		0 (0%)	1 (5%)
**Sociodemographic data**			
Employment status	20/20		
Full time employment		11 (55%)	12 (60%)
Part time employment		3 (15%)	4 (20%)
Full time education		1 (5%)	0
Part time education		1 (5%)	1 (5%)
Unemployed and actively looking for work		0	0
Unemployed and not actively looking for work		4 (20%)	3 (15%)
Retired		0	1 (5%)
Other		0	2 (10%)
Highest level of education	20/20		
Primary school		1 (5%)	0
Secondary school		6 (30%)	3 (15%)
College/undergraduate degree		3 (15%)	6 (30%)
Postgraduate certificate/diploma		3 (15%)	5 (25%)
Postgraduate degree – Masters/PhD/MBA		7 (35%)	6 (30%)
Apprenticeship		0	0
Other		0	0
**Biomedical data**			
Diabetes duration (years)	20/20	16.0 (8.2–30.8)	24.5 (9.0–36.8)
HbA1c (mmol/mol)	20/20	71.2 (25.8)	80.0 (31.2)
HbA1c (%)	20/20	8.7 (2.4)	9.5 (2.9)
BMI (kg/m^2^)	20/20	26.3 (6.0)	28.1 (6.5)
TIR (%)	19/19	49.0 (25.1)	43.7 (28.8)
CGM Wear (%)	19/17	85.0 (68.0–99.0)	75.0 (59.5–99.0)

Mental health diagnoses per DSM-5	N/N	STEADY	Control
Eating disorder	20/20	20 (100%)	20 (100%)
Anorexia nervosa		0 (0%)	0 (0%)
Bulimia nervosa		7 (35%)	7 (35%)
Binge eating disorder		0 (0%)	2 (10%)
Other Specified Feeding or Eating Disorder (OSFED)		13 (65%)	11 (55%)
Mood disorder	20/20	10 (50%)	9 (45%)
Psychotic disorder	20/20	0 (0%)	0 (0%)
Anxiety disorder	20/20	9 (45%)	6 (30%)
Trauma and trauma related disorder	20/20	1 (5%)	1 (5%)
Other DSM-5 diagnosis	20/20	7 (35%)	4 (20%)

Diabetes complications acute	N/N	STEADY	Control
**Diabetic ketoacidosis (DKA)**	20/20		
Previous DKA (lifetime)		12 (60%)	12 (60%)
Number of DKAs (lifetime) (Median and IQR)		6 (1.75–10)	1 (1–4.5)
DKA In Last 12 months		1 (5%)	0 (0%)
**Severe hypoglycaemia (SH)**			
Previous SH (lifetime)	20/20	9 (45%)	12 (60%)
Number of SHs (lifetime) (Median and IQR)	20/20	1.5 (0–4)	1 (0–4.25)
SH in last 12 months	20/20	0 (0%)	1 (5%)

Diabetes complications chronic	N/N	STEADY	Control
**Retinopathy**	20/19		
No Retinopathy		11 (55%)	7 (35%)
Background Retinopathy		6 (30%)	8 (40%)
Treated Retinopathy (Laser or Injection)		3 (15%)	4 (20%)
**Neuropathy**	20/20		
No Neuropathy		14 (70%)	14 (70%)
Tingling or Pain		6 (30%)	5 (25%)
Loss of Sensation		0 (0%)	3 (15%)
Previous or Current Ulceration		0 (0%)	1 (5%)
**Nephropathy**	20/20		
No nephropathy		20 (100%)	18 (90%)
Microalbuminuria		0 (0%)	1 (5%)
Proteinuria		0 (0%)	1 (5%)
**Cardiovascular disease**	20/20		
Angina		0 (0%)	1 (5%)
Heart Failure		0 (0%)	1 (5%)
**Autonomic Neuropathy**	20/20		
No Autonomic Neuropathy		13 (65%)	13 (65%)
Resting Tachycardia		2 (10%)	2 (10%)
Gustatory Sweating		0 (0%)	1 (5%)
Gastroparesis		1 (5%)	2 (10%)
Altered Bowel Habit		1 (5%)	1 (5%)

Person-reported outcome measures (PROM)	N/N	STEADY	CONTROL
**DEPS-R**	20/20	52.6 (16.0)	55.1 (16.3)
DEPS-R Score ≥20		20 (100%)	19 (95%)
**GAD-7**	20/20	10.6 (4.7)	10.4 (5.6)
**PHQ-9**	20/20	14.4 (5.7)	12.4 (5.8)
**EDE-QS** (median IQR)	20/20	21 (16, 25)	21 (17, 23)
EDE-QS score ≥15		17 (85%)	17 (85%)
**HFS-II** behaviour subscale	20/20	38.0 (8.3)	35.0 (9.3)
**HFS-II** worry subscale	20/20	47.7 (13.7)	48.6 (17.7)
**YBC-EDS-SRQ** preoccupations subscale	20/20	17.1 (4.0)	17.3 (4.0)
**DIDP**	20/20	2.65 (0.85)	2.34 (0.69)
**BIS/BAS** inhibition subscale	20/20	10.05 (2.37)	11.30 (4.39)
BIS/BAS reward subscale	20/20	9.10 (2.55)	9.30 (2.66)
BIS/BAS drive subscale	20/20	10.30 (3.10)	10.30 (2.60)
BIS/BAS fun seeking subscale	20/20	8.90 (2.02)	9.20 (2.67)
**T1-DDS** total	20/20	3.08 (0.88)	3.16 (0.88)
T1-DDS powerlessness subscale	20/20	4.11 (1.11)	4.10 (1.06)
T1-DDS management distress subscale	20/20	3.50 (1.59)	3.60 (1.60)
T1-DDS hypo distress subscale	20/20	2.16 (0.82)	2.54 (1.38)
T1-DDS negative social perception subscale	20/20	2.34 (1.13)	2.58 (1.47)
T1-DDS eating distress	20/20	4.62 (1.31)	4.88 (1.13)
T1-DDS physician distress	20/20	2.84 (1.48)	2.64 (1.32)
T1-DDS friend and family distress	20/20	2.11 (1.02)	1.99 (1.15)
**WHOQOL** physical health	20/20	51.7 (21.6)	48.0 (15.1)
WHOQOL psychological health	20/20	39.0 (7.9)	39.5 (7.9)
WHOQOL social health	20/20	52.6 (23.1)	61.4 (23.6)
WHOQOL environmental health	20/20	65.5 (14.0)	62.4 (20.0)

Data are n (%), median (IQR), mean (SD), or n/N (%).

HbA1c = glycated haemoglobin A1c; BMI = body-mass index; TIR = glucose time in range measured with flash- or continuous glucose monitoring; MDI = multiple daily injection therapy; CSII = continuous subcutaneous insulin infusion pump DEPS-R = Diabetes Eating Problems Survey Revised; PHQ-9 = Patient Health Questionnaire; GAD-7 = Generalised Anxiety Questionnaire; EDE-QS = Eating Disorder Examination Questionnaire Short; HFS-II = Hypoglycaemia Fear Survey; YBC-EDS-SRQ = Yale-Brown-Cornell Eating Disorder Scale Self-Report Questionnaire; DIDP = DAWN2 Impact of Diabetes Profile; BIS/BAS = Behavioural Inhibition/Behavioural Activation Scales; T1-DDS = Diabetes Distress Screening Scale for Adults with Type 1 Diabetes; WHOQOL = World Health Organization Quality of Life Assessment.

The primary outcome, the feasibility of the delivery of STEADY, was met successfully. Of the 98 participants screened, 40 were eligible and randomised (Fig. 1) (recruitment rate: 40.81%; 95% CI: 31.60%, 50.72%), with a drop-out rate of 12.5% (95% CI: 4.99%, 26.58%)–15% (4.39%, 36.55%) in the STEADY group and 10% (1.57%, 31.32%) in the control group at 6 months (Fig. 1 and Supplemental Table S3, Appendix page 5).

Secondary analyses of biomedical outcomes in the intention-to-treat analysis are presented in Table 2, Supplemental Tables S4, S6, and S7 (Appendix, page 6, 8–9). HbA1c did not differ between groups (Table 2). There was one DKA admission in each group and no severe hypoglycaemia episodes (Supplemental Table S4, Appendix, page 6). Subgroup analyses of participants wearing CGMs for >70% of the time over the 28 days prior to baseline and end-of-study assessments showed no differences between groups at baseline (Supplemental Table S6, Appendix, page 8). Secondary analyses of PROM outcomes in the intention-to-treat analyses are presented in Table 2. Participants in the STEADY group had significantly lower GAD-7 scores at follow-up compared to the control group, indicating lower anxiety, as well as higher DIDP scores, p = 0.020 indicating higher diabetes-specific quality of life (Table 2). Effect sizes were high in GAD-7 and DIDP. Baseline characteristics of participants for whom paired TIR data were available are presented in Supplemental Table S6 (Appendix, page 8).

**Table 2:** Secondary outcomes of the intention-to-treat population of the STEADY trial at study end.

	Data available in n STEADY/n Control	STEADY	Control	Mean difference (95% CI)	p-value	Effect size Cohen's d	Effect size 95% CI
**Secondary outcomes biomedical**							
HbA1c (mmol/mol)	20/19	72.9 (29.8)	76.7 (29.9)	3.86 (−15.5 to 23.24)	0.63	0.16	−0.48 to 0.79
HbA1c (%)	20/19	8.8 (2.7)	9.2 (2.7)	0.36 (−1.4 to 2.13)	0.62	0.16	−0.48 to 0.80
BMI (kg/m^2^)	14/16	26.1 (7.1)	27.0 (5.2)	0.93 (−3.77 to 5.62)	0.69	0.15	−0.57 to 0.87
TIR (%)	15/12	48 (29.9)	41.3 (31.5)	−14.5 (−44.14 to 15.20)	0.58	−0.22	−0.98 to 0.55
**Secondary outcomes PROMs**							
DEPS-R	16/16	39.3 (14.8)	47.6 (18.3)	8.31 (−3.69, 20.32)	0.17	0.50	−0.21 to 1.20
DEPS-R Score ≥20		16 (100%)	16 (100%)				
GAD-7	16/17	5.75 (2.89)	10.18 (5.31)	4.42 (1.39, 7.47)	0.0060	1.03	0.29–1.75
PHQ-9	16/17	7.69 (5.16)	10.71 (6.59)	3.018 (−1.20, 7.24)	0.16	0.51	−0.19 to 1.20
EDE-QS (median (IQR)) ∗nonparametric test, Mann–Whitney U test	16/16	9.5 (5.5, 18.3)	17 (12.5, 22.5)	−1.92	0.054	0.25	
EDE-QS score ≥15	16/16	N (%) 6 (38)	N (%) 11 (69)				
HFS-II behaviour subscale	16/16	30.25 (8.24)	35.00 (8.41)	4.75 (−1.26, 10.76)	0.18	0.57	−0.14 to 1.27
HFS-II worry subscale	16/16	36.25 (14.82)	43.94 (20.27)	7.68 (−5.13, 20.51)	0.23	0.43	−0.27 to 1.13
YBC-EDS-SRQ preoccupations subscale	16/16	17.81 (4.96)	17.56 (5.93)	−0.25 (−4.19, 3.69)	0.89	−0.05	−0.74 to 0.65
DIDP	16/16	3.13 (0.63)	2.46 (0.87)	−0.66 (−1.21, −0.11)	0.020	−0.87	−1.59 to −0.13
BIS/BAS inhibition subscale	16/16	11.69 (4.08)	12.19 (5.55)	0.5 (−3.02, 4.02)	0.77	0.103	−0.59 to 0.80
BIS/BAS reward subscale	16/16	8.56 (2.42)	8.88 (2.45)	0.31 (−1.45, 2.07)	0.72	0.13	−0.57 to 0.82
BIS/BAS drive subscale	16/16	10.06 (2.91)	10.19 (2.23)	0.12 (−1.75, 1.99)	0.89	0.05	−0.65 to 0.74
BIS/BAS fun seeking subscale	16/16	8.63 (2.36)	8.44 (2.16)	−0.18 (−1.82, 1.45)	0.82	−0.08	−0.78 to 0.61
T1-DDS total	16/16	2.26 (0.77)	2.71 (0.88)	0.44 (−0.16, 1.05)	0.14	0.54	−0.17 to 1.24
T1-DDS powerlessness subscale	16/16	3.09 (1.31)	3.61 (1.16)	0.52 (−0.37, 1.42)	0.24	0.42	−0.28 to 1.12
T1-DDS management distress subscale	16/16	2.44 (1.31)	2.84 (0.48)	0.41 (−0.60, 1.41)	0.42	0.29	−0.41 to 0.98
T1-DDS hypo distress subscale	16/16	1.80 (0.82)	2.22 (1.08)	0.42 (−0.26, 1.11)	0.22	0.44	−0.26 to 1.14
T1-DDS negative social perception subscale	16/16	1.80 (0.68)	2.47 (1.37)	0.67 (−0.12, 1.47)	0.093	0.62	−0.10 to 1.33
T1-DDS eating distress	16/16	3.10 (1.55)	4.19 (1.54)	1.083 (−0.03, 2.19)	0.056	0.70	−0.02 to 1.41
T1-DDS physician distress	16/16	2.09 (1.29)	2.09 (1.21)	0 (−0.90, 0.90)	1.00	0.00001	−0.70 to 0.70
T1-DDS friend and family distress	16/16	1.53 (0.93)	1.69 (0.70)	0.15 (−0.44, 0.75)	0.59	0.19	−0.51 to 0.881
WHOQOL physical health	16/17	56.82 (15.62)	53.88 (20.94)	−2.93 (−16.12, 10.25)	0.65	−0.16	−0.84 to 0.53
WHOQOL psychological health	16/17	47.00 (11.18)	40.53 (10.27)	−6.47 (−14.09, 1.15)	0.093	−0.60	−1.30 to 0.10
WHOQOL social health	16/17	62.13 (25.00)	64.35 (21.51)	2.22 (−14.29, 18.76)	0.79	0.096	−0.58 to 0.77
WHOQOL environmental health	16/17	74.31 (13.077)	67.41 (14.89)	6.90 (−16.88, 3.07)	0.17	−0.49	−1.18 to 0.21

Data are n (%), median (IQR), mean (SD), or n/N (%). HbA1c = glycated haemoglobin A1c; BMI = body-mass index; TIR = glucose time in range measured with flash- or continuous glucose monitoring; PROM = person-reported outcome measure; DEPS-R = Diabetes Eating Problems Survey Revised; GAD-7 = Generalised Anxiety Questionnaire; PHQ-9 = Patient Health Questionnaire EDE-QS = Eating Disorder Examination Questionnaire Short; HFS-II = Hypoglycaemia Fear Survey; YBC-EDS-SRQ = Yale-Brown-Cornell Eating Disorder Scale Self-Report Questionnaire; DIDP = DAWN2 Impact of Diabetes Profile; BIS/BAS = Behavioural Inhibition/Behavioural Activation Scales; T1-DDS = Diabetes Distress Screening Scale for Adults with Type 1 Diabetes; WHOQOL = World Health Organization Quality of Life Assessment. Data not available for all randomised patients, data given for participants with end of study data available at 6 months follow up.

Comparisons between baseline and follow-up biomedical data and PROM for each group are presented in Tables 3 and 4. Biomedical outcomes did not differ. Participants in the STEADY group (Table 3) had improved scores at study-end compared to baseline, with medium to high effect sizes in eating disorders scores (DEPS-R, EDE-QS), in anxiety and depression measures (GAD-7, PHQ-9) and the Hypoglycemia Fear Survey subscales (HFS-II-Behaviour and HFS-II Worry) (Table 3) (not reaching statistical significance due to sample size). Improvements were also seen in total T1DDS and its subscales (powerlessness, management distress, eating distress, physician distress and family and friends) as well as in the World Health Organisation Quality Of Life (WHOQOL) physical, psychological, environmental, and social health subscales (Table 3). Participants in the control group had lower scores at study-end compared to baseline with medium effect sizes in DEPS-R, total T1DDS score, and T1DDS physician distress subscale (Table 4) (not reaching statistical significance due to sample size).

**Table 3:** Comparison of secondary biomedical outcomes and person reported outcome measures (PROMs) from baseline to end of study in the STEADY group.

	N	STEADY baseline mean (SD)	STEADY end mean (SD)	Paired mean differences (95% CI)	p value	Effect size (Cohen's d)	Effect size 95% CI
**Secondary outcomes biomedical**							
HbA1c (mmol/mol)	20	71.2 (25.8)	72.9 (29.8)	1.62 (−3.88, 7.12)	0.55	−0.04	−0.30 to 0.58
HbA1c (%)	20	8.7 (2.36)	8.8 (2.7)	0.13 (−0.37, 0.64)	0.58	−0.13	−0.32 to 0.56
BMI (kg/m^2^)	14	25.7 (6.3)	26.1 (7.1)	−0.46 (−1.88, 0.96)	0.50	−0.19	−0.71 to 0.35
TIR (%)	11	47.9 (25.2)	46.2 (31.3)	−1.73 (−9.99, 6.53)	0.65	−0.14	−0.73 to 0.46
**Secondary outcomes PROMs**							
DEPS-R	16	51.94 (17.47)	39.25 (14.82)	12.68 (3.77, 21.60)	0.0084	0.76	0.19–1.31
GAD-7	16	10.81 (4.85)	5.75 (2.89)	5.06 (2.34, 7.78)	0.0012	0.99	0.38–1.58
PHQ-9	16	14.63 (5.16)	7.69 (5.16)	6.93 (3.57, 10.29)	0.00052	1.10	0.46–1.71
EDE-QS (median, IQR)	16	20 (16.25)	9.5 (5.5, 18.25)	−2.77	0.0057	−0.70	
HFS behaviour subscale	16	38.31 (8.23)	30.25 (8.24)	8.06 (3.82, 12.29)	0.0010	1.02	0.40–1.61
HFS worry subscale	16	48.44 (14.62)	36.25 (14.82)	12.18 (4.12, 20.25)	0.0057	0.81	0.23–1.36
YBC-EDS-SRQ preoccupations subscale	16	16.38 (3.83)	17.81 (4.96)	−1.43 (−4.05, 1.18)	0.26	−0.29	−0.80 to 0.21
DIDP	16	2.65 (0.85)	3.13 (0.63)	−0.47 (−1.09, 0.15)	0.13	−0.40	−0.91 to 0.11
BIS/BAS inhibition subscale	16	10.19 (2.64)	11.69 (4.08)	−1.5 (−4.29, 1.29)	0.27	−0.29	−0.78 to 0.22
BIS/BAS reward subscale	16	9.38 (2.73)	8.56 (2.41)	0.81 (−0.16, 1.79)	0.097	0.44	−0.08 to 0.95
BIS/BAS drive subscale	16	10.5 (3.18)	10.06 (2.91)	0.43 (−0.66, 1.53)	0.41	0.21	−0.28 to 0.70
BIS/BAS fun seeking subscale	16	8.88 (1.86)	8.63 (2.36)	0.25 (−0.73, 1.23)	0.59	0.14	−0.36 to 0.63
T1-DDS total	16	3.10 (0.74)	2.26 (0.77)	0.83 (0.45, 1.22)	0.00031	1.16	0.51–1.79
T1-DDS powerlessness subscale	16	4.08 (1.18)	3.09 (1.31)	0.98 (0.33, 1.63)	0.0055	0.81	0.23–1.37
T1-DDS management distress subscale	16	3.63 (1.68)	2.44 (1.31)	1.18 (0.55, 1.81)	0.0011	1.00	0.391–1.60
T1-DDS hypo distress subscale	16	2.20 (0.90)	1.80 (0.82)	0.41 (−0.017, 0.82)	0.059	0.51	−0.02 to 1.03
T1-DDS negative social perception subscale	16	2.39 (1.25)	1.80 (0.68)	0.59 (−0.13, 1.31)	0.10	0.44	−0.08 to 0.94
T1-DDS eating distress	16	4.56 (1.35)	3.10 (1.54)	1.45 (0.72, 2.19)	0.00072	1.06	0.43–1.67
T1-DDS physician distress	16	2.88 (1.51)	2.09 (1.29)	0.78 (0.36, 1.19)	0.0011	1.01	0.39–1.60
T1-DDS friend and family distress	16	2.09 (1.02)	1.53 (0.92)	0.56 (0.19, 0.93)	0.0054	0.813	0.23–1.37
WHOQOL physical health	16	48.6 (15.4)	56.8 (15.6)	−8.18 (−13.53, −2.83)	0.0053	−0.82	−1.37 to −0.24
WHOQOL psychological health	16	39.3 (8.4)	47 (11.2)	−7.75 (−14.01, −1.49)	0.019	−0.66	−1.19 to −0.11
WHOQOL social health	16	53.3 (24.4)	62.1 (25.0)	−8.87 (−16.72, −1.02)	0.029	−0.60	−1.13 to −0.06
WHOQOL environmental health	16	64.6 (12.1)	74.3 (13.11)	−9.75 (−16.13, −3.36)	0.0054	−0.81	−1.37 to −0.23

Data are n (%), median (IQR), mean (SD), or n/N (%). HbA1c = glycated haemoglobin A1c; BMI = body-mass index; TIR = glucose time in range measured with flash- or continuous glucose monitoring; MDI = multiple daily injection therapy; CSII = continuous subcutaneous insulin infusion pump DEPS-R = Diabetes Eating Problems Survey Revised; GAD-7 = Generalised Anxiety Questionnaire; PHQ-9 = Patient Health Questionnaire; EDE-QS = Eating Disorder Examination Questionnaire Short; HFS-II = Hypoglycaemia Fear Survey; YBC-EDS-SRQ = Yale-Brown-Cornell Eating Disorder Scale Self-Report Questionnaire; DIDP = DAWN2 Impact of Diabetes Profile; BIS/BAS = Behavioural Inhibition/Behavioural Activation Scales; T1-DDS = Diabetes Distress Screening Scale for Adults with Type 1 Diabetes; WHOQOL-BREF = World Health Organization Quality of Life Assessment. Data not available for all randomised patients, data given for participants with end of study data available at 6 months follow up.

**Table 4:** Comparison of secondary biomedical outcomes and person-reported outcome measures (PROM) from baseline to end of study in the control group.

	N	Mean control baseline (SD)	Mean control end (SD)	Paired mean differences (95% CI)	p value	Effect size (Cohen's d)	Effect size 95% CI
**Secondary outcomes biomedical**							
HbA1c (mmol/mol)	19	80.3 (32.0)	76.7 (29.9)	3.6 (−1.81, 9.02)	0.18	0.32	−0.15 to 0.78
HbA1c (%)	19	9.5 (2.9)	9.2 (2.7)	0.33 (−0.16, 0.83)	0.18	0.32	−0.14 to 0.78
BMI (kg/m^2^)	16	27.1 (5.5)	27.0 (5.4)	0.02 (−0.41, 0.44)	0.94	0.02	−0.47 to 0.51
TIR (%)	10	30.0 (25.2)	31.7 (24.4)	1.71 (−24.77, 28.20)	0.84	0.06	−0.68 to 0.78
**Secondary outcomes PROMs**							
DEPS-R	16	55.56 (16.44)	47.56 (18.26)	8 (2.57, 13.42)	0.0067	0.79	0.21–1.34
GAD-7	17	11.3 (5.5)	10.2 (5.3)	1.12 (−1.81, 4.042)	0.43	0.20	−0.29 to 0.67
PHQ-9	17	13.4 (5.6)	10.7 (6.6)	2.64 (−0.55, 5.84)	0.099	0.43	−0.08 to 0.91
EDE-QS (median, IQR)	16	19.4 (6.3)	21.0 (17.0, 23.8)	17.0 (12.5, 22.5)	−1.89	0.059	
HFS behaviour subscale	16	34.4 (9.4)	35 (8.44)	−0.62 (−5.44, 4.19)	0.79	−0.07	−0.55 to 0.42
HFS worry subscale	16	47.4 (18.3)	43.9 (20.3)	3.43 (−6.17, 13.051)	0.46	0.19	−0.31 to 0.68
YBC-EDS-SRQ preoccupations subscale	16	17.3 (4.4)	17.6 (5.9)	−0.31 (−3.71, 3.09)	0.85	−0.049	−0.56 to 0.44
DIDP	16	2.4 (0.7)	2.5 (0.9)	−0.12 (−0.57, 0.32)	0.56	−0.15	−0.64 to 0.35
BIS/BAS inhibition subscale	16	11.3 (4.6)	12.2 (5.6)	−0.93 (−2.012, 0.13)	0.08	−0.47	−0.98 to 0.06
BIS/BAS reward subscale	16	9.6 (2.7)	8.9 (2.4)	0.75 (−0.42, 1.92)	0.19	0.34	−0.17 to 0.84
BIS/BAS drive subscale	16	10.6 (2.8)	10.2 (2.3)	0.37 (−0.32, 1.073)	0.27	0.29	−0.22 to 0.78
BIS/BAS fun seeking subscale	16	9.4 (2.9)	8.4 (2.2)	1 (−0.27, 2.27)	0.12	0.42	−0.10 to 0.92
T1-DDS total	16	3.12 (0.97)	2.71 (0.89)	0.39 (0.055, 0.74)	0.026	0.618	0.073–1.146
T1-DDS powerlessness subscale	16	4.04 (1.16)	3.61 (1.16)	0.42 (−0.075, 0.92)	0.090	0.453	−0.069 to 0.962
T1-DDS management distress subscale	16	3.43 (1.68)	2.84 (1.48)	0.59 (−0.18, 1.37)	0.12	0.407	−0.109 to 0.912
T1-DDS hypo distress subscale	16	2.45 (1.38)	2.22 (1.08)	0.23 (−0.32, 0.79)	0.39	0.224	−0.276 to 0.717
T1-DDS negative social perception subscale	16	2.48 (1.63)	2.47 (1.37)	0.016 (−0.42, 0.46)	0.94	0.019	−0.471 to 0.508
T1-DDS eating distress	16	4.91 (1.13)	4.18 (1.54)	0.72 (−0.0083, 1.46)	0.052	0.527	−0.005 to 1.043
T1-DDS physician distress	16	2.59 (1.30)	2.09 (1.21)	0.5 (0.11, 0.89)	0.016	0.679	0.124–1.216
T1-DDS friend and family distress	16	2.05 (1.25)	1.69 (0.70)	0.35 (−0.22, 0.94)	0.21	0.329	−0.179 to 0.827
WHOQOL physical health	17	46.5 (10.9)	53.9 (20.9)	−7.35 (−16.29, 1.58)	0.10	−0.42	−0.91 to 0.08
WHOQOL psychological health	17	39.5 (7.95)	40.5 (10.27)	−1.059 (−5.97, 3.85)	0.65	−0.11	−0.59 to 0.37
WHOQOL social health	17	59.29 (23.50)	64.35 (21.51)	−5.059 (−11.24, 1.13)	0.10	−0.42	−0.91 to 0.08
WHOQOL environmental health	17	60.88 (20.21)	67.41 (14.90)	−6.52 (−13.07, 0.020)	0.051	−0.51	−1.01, 0.01

Data are n (%), median (IQR), mean (SD), or n/N (%). HbA1c = glycated haemoglobin A1c; BMI = body-mass index; TIR = glucose time in range measured with flash- or continuous glucose monitoring; MDI = multiple daily injection therapy; CSII = continuous subcutaneous insulin infusion pump; DEPS-R = Diabetes Eating Problems Survey Revised; GAD-7 = Generalised Anxiety Questionnaire; PHQ-9 = Patient Health Questionnaire; EDE-QS = Eating Disorder Examination Questionnaire Short; HFS-II = Hypoglycaemia Fear Survey; YBC-EDS-SRQ = Yale-Brown-Cornell Eating Disorder Scale Self-Report Questionnaire; DIDP = DAWN2 Impact of Diabetes Profile; BIS/BAS = Behavioural Inhibition/Behavioural Activation Scales; T1-DDS = Diabetes Distress Screening Scale for Adults with Type 1 Diabetes; WHOQOL-BREF = World Health Organization Quality of Life Assessment. Data not available for all randomised patients, data given for participants with end of study data available at 6 months follow up.

Adverse event data are provided in Supplemental Table S4 (Appendix, page 6). No adverse events or serious adverse events were related and unexpected to the study intervention. Six severe adverse events were reported in the STEADY arm, including one intracranial bleed (after the therapy had ended, spontaneous subarachnoid bleed), one hospital admission for an unrelated medical issue (gallstones), two hospital admissions for hyperglycaemia, and one acute mental health episode (psychosis, requiring several hospital admissions). Data on use of health care systems for diabetes and mental health are provided in Supplemental Tables S8 and S9 (Appendix, pages 9–10).

## Discussion

The feasibility randomised controlled trial of the STEADY intervention tested the first complex intervention combining diabetes education and CBT for T1DE. The intervention manual, co-designed with people with lived experience and multidisciplinary healthcare professionals, is the first that targets eating disorder behaviours and cognitions while fully integrating diabetic medicine and the broader context of how living with T1D impacts on all mental health domains.

The delivery of STEADY was feasible in terms of recruitment, delivery, and retention of participants in the intervention, with drop-out rates comparable to and lower than previous complex intervention trials in diabetes and mental health that demonstrated 17%, 44% and 44% attrition rates, respectively.^25^^,^^26^ Secondary outcomes analyses demonstrated proof-of-concept that mental health domains related to diabetes can be improved with this intervention, including improvements in eating disorders cognitions and behaviours, anxiety, depression, and quality of life related to and unrelated to living with T1D, without deteriorating glycaemic control or causing adverse events related to the intervention. For the definitive trial, we will aim to include person reported outcome measures in addition to biomedical outcomes, which in combination with the attrition rates from this feasibility trial will inform the power calculations.

This is the first intervention tailored to adults with established T1DE that fully integrates diabetes-related mental and physical health, delivered by a diabetes healthcare professional using CBT methods. This is in contrast to generic eating disorder interventions^4^^,^^23^^,^^27^ which did not integrate diabetes-specific psychological and medical factors. Interventions for the primary prevention of disordered eating^28^^,^^29^ are conceptually different from the treatment of disordered eating in T1D: The STEADY intervention aims to treat people with T1DE that presents in thoughts, feelings, cognitions and behaviour impacting on diabetes selfcare and/or disordered eating behaviour, adversely impacting on health and wellbeing. In the context of the STEADY intervention for T1DE, the cognitions and affect are linked to behaviours, therefore we were targeting all three. In primary prevention, the behaviour (e.g., insulin omission with the intention to control weight, or significant food restriction leading to problematic hypoglycaemia or weight loss) has not (in theory) manifested yet. Applying elements of the STEADY intervention as a preventative measure would be different in nature-it would require a different set up, e.g., recruitment early after diabetes diagnosis, perhaps in a group format (to make it less cost intense) and the success would be measured in a longitudinal study measuring rates of people developing T1DE. In future, elements of STEADY could be adjusted for a more preventative approach.

Although STEADY was designed for mild to moderate T1DE severity, over 70% of the participants in this trial had one or more additional mental disorder, emphasising the need to integrate mental health problems linked with T1D. This aligns with our work with the severe T1DE clinical service patients, where over two-thirds had additional mental disorders and required a high intensity multidisciplinary approach.^5^ STEADY is an outpatient-based intervention delivered remotely over a maximum of 12 sessions in this instance, making it less resource-intensive than treatments for severe T1DE,^5^ and more suitable for those with mild to moderate spectrum T1DE who have lower acute medical risk and who may prefer an intervention requiring less travel time and time off work.

Unlike existing T1DE interventions, STEADY was co-designed by people with lived experience of T1DE, following a stepwise process of EBCD,^6^ integrating findings of focus groups and interviews,^10^ development of theoretical models,^2^^,^^9^ and mixed methods approaches to understand the interplay between disordered eating and T1D physiology.^30^

Having a modular therapy toolkit allowed the therapist to choose elements specific for each participant, and tailor therapy to the formulation. This differs from other manualised interventions^31^^,^^32^ found in eating disorder therapies and psychoeducational interventions targeting diabetes distress^33^ or problematic hypoglycaemia.^34^ This strength of the tailored STEADY toolkit approach is reflected in the secondary PROM mental health outcomes which improved across the board in the intervention group, and significantly more in anxiety and diabetes specific quality of life measures.

Another strength is the delivery of STEADY in a virtual/remote modality, supported by an optional app to facilitate CBT exercises and communication. STEADY can be delivered in addition to usual diabetes care by diabetes specialists trained in the STEADY-CBT approach, allowing a better geographical reach of this intervention whilst patients remain embedded in their usual care settings. This is important for a time-limited intervention for a chronic and life-long condition and avoids disruptions of moving care between teams. The experience of having a multidisciplinary team who is knowledgeable and trained in T1DE seemed to have a positive impact, as physician-related diabetes distress reduced compared to baseline in both groups.

A limitation of this trial, being a feasibility RCT, is that it was not designed to be powered to detect differences in HbA1c, other biomedical outcomes measures and person reported outcomes measures. We included PROMs from both research areas (diabetes and eating disorders) as part of the feasibility study, in order to understand which ones are the best suited to be included in the definitive trial, which limits the interpretation of statistical within condition findings. Participants with a broad spectrum of HbA1c values were included due to the many different presentations of T1DE, which precluded subgroup analyses. For example, we intentionally included participants with HbA1c outcomes within clinical target range who met T1DE criteria, as they were achieved through restrictive eating patterns and with high levels of anxiety and perfectionism; we also included participants who were using new diabetes technologies and met clinical targets for TIR, but struggled with binge eating disorder. Due to limited sample size, a subgroup analysis was not conducted. This is in contrast to severe T1DE, where all participants fulfilled biomedical severity criteria, one of which being HbA1c > 10%, where we detected a reduction in Hb1Ac.^5^ Another limitation is that we were not able to test whether face-to-face delivery would have made a difference compared to virtual delivery modality, as the trial commenced during the COVID-19 pandemic and had required adjustments adjusted to all-virtual delivery. Compared to the background population of people with type 1 diabetes, the distribution of ethnicity in our sample was representative,^35^ it is also known that eating disorders are more prevalent in women and that men tend to engage less in eating disorder research. We will plan an inclusive and diverse recruitment strategy to ensure that groups who are less likely to access eating disorder research take part in the definitive trial.^36^

The next steps include optimising the therapy manual, testing STEADY in a full-scale multi-centre RCT, and formalising STEADY-CBT training to increase its accessibility for diabetes specialists and eating disorders healthcare professionals in view of broader implementation within diabetes healthcare services.

## Contributors

MS drafted the report; MS, NZ, AH, DP and JB were responsible for intervention development and content; NZ and MS were responsible for trial conduct; DH, JT and KI provided input to the trial design and oversight of trial conduct; JA and NZ led on patient and public involvement; EK coordinated the development of the smartphone app; JB and AH delivered the intervention, AH and MS provided clinical supervision; DP and AH conducted the SCID assessments; NZ, MS, AH and DP were collecting data; RT collected CGMS data and supported statistical analysis; SA and MS were responsible of the statistical analyses, NZ and RT contributed to the statistical analyses. All authors had full access to all the data in the study, reviewed the final version of the manuscript and had final responsibility for the decision to submit for publication. MS, NZ and RT have accessed and verified the underlying data reported here.

## Data sharing statement

Deidentified participant data will be made available on request (e.g., for meta-analyses) to the investigators, the study protocol has been previously published. The STEADY intervention manual is intellectual property of King's College London and is currently being refined and converted into a short course university module-it cannot be shared at present.

## Declaration of interests

We declare no competing interests.

## Appendix A Supplementary data

Protocol Steady
