# Supplementary material for: Safety of a co-designed cognitive behavioural therapy intervention for people with type 1 diabetes and eating disorders (STEADY): a feasibility randomised controlled trial
Source: Lancet Reg Health Eur. 2025 Jan 20;50:101205. doi: 10.1016/j.lanepe.2024.101205 (PMC11788855; doi:10.1016/j.lanepe.2024.101205)
Supplement: Protocol Steady [file mmc1.pdf]

Check for updates

Received: 26 September 2023 | Accepted: 9 December 2023

DOI: 10.1111/dme.15273

STUDY PROTOCOLS

DIABETIC Medicine

Protocol for the STEADY intervention for type 1 diabetes and disordered eating: Safe management of people with Type 1 diabetes and EAting Disorders studY

Natalie Zaremba[1] | Amy Harrison[1,2,3] | Jennie Brown[1,4] | Jacqueline Allan[1] | Divina Pillay[1] | Janet Treasure[5] | Salma Ayis[6] | David Hopkins[7,8] | Khalida Ismail[2] | Marietta Stadler[1,2]

[1]Department of Diabetes, School of Cardiovascular and Metabolic Medicine and Sciences, King's College London, London, UK

[2]Department of Psychological Medicine, Diabetes, Psychology and Psychiatry Research Group, King's College London, London, UK

[3]Department of Psychology and Human Development, Institute of Psychiatry, University College London, London, UK

[4]Diabetes Centre, King's College Hospital, London, UK

[5]Institute of Psychiatry, Psychology and Neuroscience, King's College London, London, UK

[6]School Population Health and Environmental Sciences, King's College London, London, UK

[7]Department of Diabetes, School of Life Course Sciences, King's College London, London, UK

[8]Institute of Diabetes, Endocrinology and Obesity, King's Health Partners, London, UK

Correspondence
Marietta Stadler, Department of Diabetes, School of Cardiovascular and Metabolic Medicine and Sciences, King's College London, London, UK.
Email: marietta.stadler@kcl.ac.uk

Funding information
National Institute for Health and Care Research

Abstract

This paper describes the protocol to test the feasibility of the Safe management of people with Type 1 diabetes and EAting Disorders studY (STEADY) intervention. STEADY is a novel complex intervention for people with type 1 diabetes and disordered eating (T1DE) of mild to moderate severity. The STEADY intervention integrates cognitive behavioural therapy (CBT) with diabetes education, and was developed using Experience-Based Co-Design.

Methods: The feasibility of STEADY will be tested using a randomised controlled feasibility trial. Forty adults with T1DE will be recruited and randomised into the STEADY intervention or treatment as usual control group. We will collect demographic, biomedical and psychometric data, routine glucose metrics and conduct the Structured Clinical Interview for DSM-5. Participants randomised to the STEADY intervention will receive 12 STEADY therapy sessions with a diabetes specialist nurse trained in CBT, delivered via videoconference and an optional smartphone app. The main outcome at 6 months will be the feasibility of STEADY (recruitment, dropout rates, feasibility of delivery). The secondary outcomes are biomedical (HbA1c and glucose time in range) and psychological (person-reported outcome measures in disordered eating, diabetes distress, depression and anxiety). A process evaluation will evaluate the fidelity, feasibility, acceptability and appropriateness of STEADY, and participant experiences.

Ethics and dissemination: The protocol was approved by the East of England—Essex Research Ethics Committee (21/EE/0235). Study findings will be shared with study participants and disseminated through peer-reviewed publications and conference presentations.

KEYWORDS

cognitive behaviour therapy, eating disorder, intervention, process evaluation, T1DE, type 1 diabetes

Clinical Trials Registry No; ClinicalTrials.gov NCT05140564

 © 2024 Diabetes UK.

1 | INTRODUCTION

Type 1 diabetes (T1D) and disordered eating (T1DE) is a complex and dangerous comorbidity for which there is no evidence-based treatment that improves biomedical outcomes.[1] The prevalence of T1DE is estimated to be between 8 and 36% with an additional 9% to 14% of people with T1D having sub-threshold disordered eating, although rates may be even higher in certain populations, with a recent study finding disordered eating in 53% of Māori young adults with T1D.[2–7] T1DE is associated with an increased risk of mortality and acute and long-term complications.[8] T1DE presents with diabetes-specific disordered eating or diabetes self-care neglect behaviours that can feature in varying combinations, such as deliberate insulin omission or restriction, adopting restrictive low-carbohydrate diets to reduce insulin requirements or blood glucose fluctuations, and episodes of binge eating in response to hypoglycaemia symptoms.[9–11]

Existing eating disorder or T1D interventions are incompatible with the treatment of T1DE and do not address the complexity of both conditions, for example, T1D management interventions do not address fear of weight gain and encourage restrictive eating patterns, while eating disorder interventions do not address T1D management behaviours such as counting carbohydrates to accurately dose insulin.[1] Healthcare professional teams with experience of treating T1DE have described more effective treatment when T1D and eating disorder teams can jointly treat patients, and when healthcare providers have appropriate training and understanding in both disciplines.[12]

We developed the Safe management of people with Type 1 diabetes and EAting Disorders studY (STEADY) intervention toolkit for T1DE using the Experience-Based Co-Design (EBCD) process,[13] following the Medical Research Council guidance for developing complex interventions[14] and informed by our previously developed theoretical model of T1DE from literature reviews, a thematic analysis of blogs written by people with T1DE,[15] semi-structured interviews with people with T1DE,[16] and focus group with healthcare professional teams.[12] The resulting STEADY intervention is a novel complex intervention based ia cognitive behavioural therapy (CBT) that has been co-designed by people with lived experience of T1DE, and healthcare professionals who are experienced in treating T1DE (STEADY phase I).[13]

STEADY phase II aims to test the STEADY therapy toolkit in a feasibility randomised controlled trial (RCT) of 12 sessions of STEADY therapy compared with treatment as usual. The aim of this paper is to describe the protocol for the STEADY feasibility RCT.

What's new?

- What is already known? Current eating disorder interventions are not effective for people with type 1 diabetes and disordered eating (T1DE), and there are currently no effective evidence-based interventions.
- What this study has found? The study described in this protocol will test the feasibility of a novel complex intervention for T1DE that incorporates T1DE-specific cognitive behaviour therapy and diabetes education.
- What are the implications of the study? This is the first feasibility randomised controlled trial of an intervention tailored for people with T1DE that integrates physical and psychological health.

2 | METHODS

This study received ethical approval by the East of England—Essex Research Ethics Committee (21/EE/0235). The trial registration number is NCT05140564.

2.1 | Trial design

This is an RCT to determine the feasibility of the STEADY intervention.[13] Participants with mild to moderate T1DE randomised into the intervention group receive 12 sessions of the STEADY CBT intervention integrated with evidence-based diabetes education and self-care.[13] Participants in the control group receive their usual care from their local medical teams. The trial started in March 2022 and the expected completion date is March 2024.

2.2 | Patient and public involvement

The STEADY project has an active Patient and Public Involvement (PPI) group established in 2017, which meets twice per year to discuss aspects of the STEADY research project.

14645491, 2024, 4, Downloaded from https://onlinelibrary.wiley.com/doi/10.1111/dme.15273 by <Shibboleth>-member@kcl.ac.uk, Wiley Online Library on [18/10/2024]. See the Terms and Conditions (https://onlinelibrary.wiley.com/terms-and-conditions) on Wiley Online Library for rules of use; OA articles are governed by the applicable Creative Commons License

2.3 | Accessibility and inclusion

We have ensured that STEADY is culturally sensitive using the principles proposed by the NHS Talking Therapies Positive Practice Guide. All study materials are available digitally via smartphone app or via email, as well as paper formats that can be printed, sent via post or completed in-person. Materials are available in large text formats. Participants who do not have access to a smartphone will be provided with one to loan for the duration of the study so they may access the STEADY smartphone app if they wish, or if they require a device to access virtual therapy sessions. The use of remote consultations can lower the threshold to accessing the diabetes healthcare teams and can be cost-effective for healthcare services. People without access to videoconferencing (via computer or smartphone) are also offered face-to-face intervention delivery.

2.4 | Setting

The trial is run by King's College London, with baseline and end of study appointments taking place in King's College Hospital Clinical Trials Facility (https://ctu.co.uk/).

Therapy visits for participants in the STEADY intervention group will take place virtually over Zoom (https://zoom.us/) which was chosen for the whiteboard feature that helps facilitate CBT exercises. Virtual therapy sessions provide flexibility and safety to research participants and staff during the COVID-19 pandemic.

2.5 | Study inclusion

Forty adults with T1DE will be recruited. Key inclusion and exclusion criteria are listed in Table 1. Potential participants with high risk of severe medical outcomes will be excluded and referred to their local diabetes teams. Potential participants with more severe mental illness are unlikely to benefit from a CBT-based approach and will be excluded from the trial to not delay more appropriate treatment.

TABLE 1 Inclusion and exclusion criteria for STEADY randomised controlled trial.

**Inclusion criteria**
1. Adults (≥18 years)
2. Diagnosis of type 1 diabetes mellitus for at least 6 months
3. Current disordered eating, defined as restriction or manipulation of insulin to control weight, food restriction, binge eating, any additional disordered eating behaviour as described by the DSM-5, or ICD-11, or a score of 15 on the EDE-QS or a score of 20 on the DEPS-R
4. Prepared to take part in the STEADY intervention group (12 sessions of T1DE-specific CBT and diabetes education)
5. Prepared to take part in the treatment as usual control group
6. Prepared to attend a face-to-face physical and mental health check-up at baseline and at the end of the study
7. Currently under the care of a diabetes specialist team
8. Confirms availability to attend all sessions as part of the intervention
9. Participant has capacity to consent to the study. This is assessed by ability to explain information about their involvement in the study
**Exclusion criteria**
1. HbA1c >15% (140.4 mmol/mol)
2. More than two admissions for DKA in the past 12 months due to insulin omission (not if triggered by infection or cannula failure)
3. More than 2 severe hypoglycaemia episodes (defined as needing 3rd party assistance) in the past 12 months
4. Severe mental illness, including severe depression with suicidal ideation, psychosis, emotionally unstable personality disorder requiring more intensive psychiatric treatment, substance problem use and dependence
5. Body mass index below 15 kg/m^2
6. Significant cognitive impairment
7. Unable to speak/read/write in English
8. Unable to give written informed consent
9. Pregnant or planning pregnancy
10. Advanced diabetes complications (end-stage renal failure, registered blind, limb amputation)
11. Uncontrollable electrolyte disturbance, low blood pressure (<100/60 mmHg), ECG abnormalities related to malnutrition (QTc-prolongation) or other physical conditions requiring inpatient treatment

2.6 | Study procedures

The timeline for the study is shown in Figure 1.

2.6.1 | Recruitment

Potential participants expressing interest will be given the participant information sheet, and an opportunity to discuss the study with a member of the research team. Potential participants will have a pre-screening phone call with a trial clinician to assess eligibility and medical safety. If the participant is eligible at pre-screening and wishes to proceed, they will sign a consent form and will be invited to a baseline study visit.

2.6.2 | Baseline visit

A multidisciplinary baseline clinical assessment will be done to collect physical health, diabetes, and mental health history, HbA1c, and questionnaires for person-reported

14645491, 2024, 4, Downloaded from https://onlinelibrary.wiley.com/doi/10.1111/dme.15273 by <Shibboleth>-member@kcl.ac.uk, Wiley Online Library on [18/10/2024]. See the Terms and Conditions (https://onlinelibrary.wiley.com/terms-and-conditions) on Wiley Online Library for rules of use; OA articles are governed by the applicable Creative Commons License

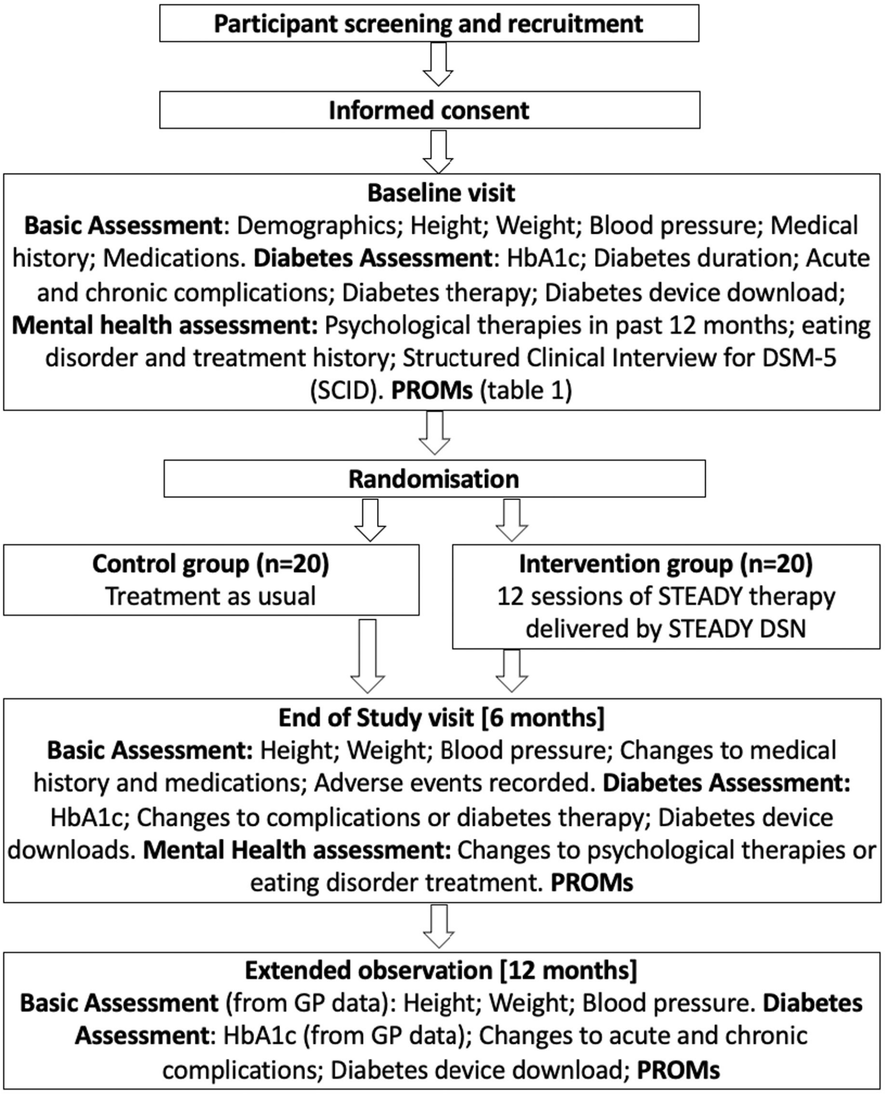

FIGURE 1 STEADY randomised controlled trial flow chart.

outcome measures (PROMS). The diabetologist, psychiatrist and clinical psychologist will jointly assess whether the participant is safe to proceed with STEADY, or whether they need signposting towards clinical treatment or therapy of a different type.

Height, weight, and resting blood pressure will be recorded. The research team will document any concomitant medications including diabetes medication. Medical and diabetes history and data (including 2-week and 3-month downloads of glucose metre, continuous (CGM) or flash glucose monitor (fCGM), insulin pump, or connected insulin pen if available) will be collected. Participants who do not use fCGM or CGM will be given a blinded Dexcom G6 continuous glucose monitor to collect blood glucose data for 10 days.

The study diabetologist will review aspects of diabetes education to ensure all participants have sufficient basic education of diabetes self-management including treatment of hypoglycaemia, sick day rules, and safe injection techniques.

The study psychiatrist or clinical psychologist will conduct a mental health assessment, eating disorder history, the Structured Clinical Interview for DSM-5,[17] and develop a 'mental health crisis plan' with the participant to ensure the participant has adequate signposting in the event of a mental health emergency, including their GP, diabetes team and mental health teams, and trusted members of family or friends they feel comfortable calling. In addition, crisis support phone and text numbers, and instructions on when to attend local urgent care are provided.

Participants will complete a set of PROMs (Table 2). All questionnaires have been validated and are routinely used in research or clinical practice.

2.6.3 | Randomisation

Participants will be randomised using 4 by 4 block sizes using the randomisation system provided by King's

14645491, 2024, 4, Downloaded from https://onlinelibrary.wiley.com/doi/10.1111/dme.15273 by <Shibboleth>-member@kcl.ac.uk, Wiley Online Library on [18/10/2024]. See the Terms and Conditions (https://onlinelibrary.wiley.com/terms-and-conditions) on Wiley Online Library for rules of use; OA articles are governed by the applicable Creative Commons License

TABLE 2 Person reported outcome measures (PROMs) asked at baseline, end of study, and 12 month extended follow up.

Questionnaire name	Psychometrics	
Patient Health Questionnaire (PHQ-9)	Standardised mood measure	[19]
Generalised Anxiety Questionnaire (GAD-7)	Standardised anxiety measure	[20]
Diabetes Distress Screening Scale for Adults with Type 1 Diabetes (T1-DDS)	Diabetes distress for T1D	[24]
Diabetes Eating Problems Survey Revised (DEPS-R)	Eating disorder behaviours in T1D	[18]
Hypoglycaemia Fear Survey (HFS-II)	Hypoglycaemia fear survey for adults with T1D	[25]
Behavioural Inhibition/Behavioural Activation Scales (BIS/BAS)	Behavioural inhibition and activation scales, measuring reward and punishment	[26]
Eating Disorder Examination Questionnaire Short (EDE-QS)	Eating disorders examination short form questionnaire used routinely in eating disorder services	[27]
DAWN2 Impact of Diabetes Profile (DIDP)	DAWN impact of diabetes profile, measures impact of T1D on quality of life	[28]
Client Services Receipt Inventory for health service use (CSRI)	Client Services Receipt Inventory, assesses service use in medical populations	[29]
World Health Organization Quality of Life Assessment (WHOQOL-BREF)	General quality of life measure	[30]
Preoccupations subscale of the Yale-Brown-Cornell Eating Disorder Scale Self-Report Questionnaire (YBC-EDS-SRQ)	Yale-Brown-Cornell Eating Disorder Scale Self-Report Questionnaire measure eating and weight-related preoccupations	[31]

Clinical Trials Unit. Participants will be notified of their group via telephone. Once a participant is randomised, they will remain in the study unless they choose to withdraw or they are withdrawn. Participants withdrawn prior to randomisation will be replaced.

The research team, participants and participants' usual care providers cannot be blinded to group allocation, as all members of the research team are part of the multidisciplinary team monitoring and ensuring participants' safety in the trial. The trial statistician will remain blinded to group allocation throughout.

STEADY intervention group

Participants in the STEADY group will receive up to 12 sessions of T1DE-specific CBT delivered by a diabetes specialist nurse (DSN). The participant's routine diabetes care will remain with their usual care teams.

Control group

Participants in the control group will continue to receive usual medical care from their local diabetes team and will be referred to their local eating disorder services as recommended by NICE.

2.6.4 | End of study visit

The end of study visit will take place 6 months after randomisation (±6 weeks). Both intervention and control groups will attend the final visit in-person. Data collection will be repeated (HbA1c, PROMs, changes to physical and mental health, medications, diabetes management or physical therapies, download of diabetes devices). Adverse events and hospitalisations will be recorded.

2.6.5 | Extended observation

A remote extended observation visit will take place 12 months after randomisation via telephone or videoconference. Blood analyses, baseline questionnaires, hospital admissions, device downloads where available will be collected. Both intervention and control groups will take part in this extended observation.

2.7 | STEADY therapy

The STEADY toolkit will be followed using a modular approach that is tailored to each participant's needs,[13] depending on the initial psychotherapeutic formulation. A key component of STEADY is behavioural experiments which involve gradual changes to diabetes management behaviour, for example, gradual titration of insulin injection at a pace that is medically safe and that is tolerable to the participant.[13] Other components of the toolkit address binge eating, food restriction, fear of hypoglycaemia, adjusting to exercise, perfectionism and acceptance of diabetes.[13]

The DSN delivering the intervention (JB) has longstanding experience in the management of T1DE and has

14645491, 2024, 4, Downloaded from https://onlinelibrary.wiley.com/doi/10.1111/dme.15273 by <Shibboleth>-member@kcl.ac.uk, Wiley Online Library on [18/10/2024]. See the Terms and Conditions (https://onlinelibrary.wiley.com/terms-and-conditions) on Wiley Online Library for rules of use; OA articles are governed by the applicable Creative Commons License

completed CBT training through an advanced postgraduate diploma in CBT. The DSN was part of the multidisciplinary team that developed the STEADY toolkit and ensured all materials in the STEADY toolkit were appropriate in the context of both CBT and diabetes education.[13] The DSN delivering STEADY will undertake regular CBT skill assessment and supervision by an experienced CBT-trained clinical psychologist (AH), psychiatrist (DP) and will have support for medical issues from the study diabetologist (MS). We will also pilot the feasibility of the inverse therapy delivery model, with the study clinical psychologist (AH) being trained in the basics of T1D and providing STEADY therapy to participants with low medical risk (no admissions in DKA or severe hypoglycaemia) under the supervision of the study DSN and diabetologist.

2.7.1 | Intervention schedule

The first four therapy sessions will be conducted weekly, the final eight sessions can be held weekly, every other week or at a schedule that suits the participant. After the fourth session, the DSN and participant will reflect on the participant's first sessions and determine if the participant benefits from continued treatment. If the participant has not felt engaged in the therapy and does not perceive benefit, they may pause therapy for a defined number of weeks agreed with the therapist. Therapy can be emotionally challenging, and difficulty engaging with therapy is common in eating disorder recovery. Therefore, this option allows participants to pause for a defined time until they feel more adequately prepared for therapy rather than withdrawing from the study. Participants are allowed to withdraw from the intervention at any time in the study if they wish and can remain in the study for follow-up data collection.

2.7.2 | Therapy session format

In the first therapy session, the participant will be introduced to the fundamental components of CBT, and the therapist will work with the participant to develop a CBT formulation.

Prior to each subsequent session, participant will complete the DEPS-R[18] as well as the PHQ-9[19] and GAD-7[20] on the STEADY smartphone app. This will allow any serious changes to diabetes distress, depression, or anxiety to be picked up by the study DSN prior to the session.

In sessions 2–11, the therapist and participant will work together towards goals set together in the session. The format for sessions will be

- A brief check containing standard items (mood, blood glucose levels, ketone levels, insulin)
- Summary of the previous session
- Agenda setting, agreeing what that session will concentrate on
- Review of the agreed 'active practice' since the last session
- Main agenda items
- Re-setting or reviewing progress towards previously agreed goals
- Setting new active practice tasks informed by the session
- A final summary, what has been learned during the session

The participants will record their CBT active practice and notes in the STEADY app or paper STEADY worksheets. The study DSN will send the participant their summary of the session on the STEADY app or via email.

Participants will also set three individualised biopsychosocial recovery goals, which include mental health, physical health and social goals, and monitor their progress on a 0–10 scale throughout the course of their therapy sessions.

The final STEADY session will summarise the participant's progress and review relapse prevention. Each participant will have an individualised CBT end of therapy plan which will include

- What I have learned in therapy?
- How will I know if I'm at risk of relapse?
- What strategies may help prevent relapse?
- If I relapse, what have I learned that may help me get back on track?

2.7.3 | Clinical supervision

The DSN delivering the STEADY intervention will receive 1.5 h of supervision from the team clinical psychologist each week. The DSN will dedicate 10 min after each session to document their reflection in real time. The supervisory sessions will be documented as a shared reflective diary at the end of each supervision session.

2.8 | STEADY app

Participants will have the option to use a bespoke STEADY smartphone app to facilitate therapy delivery and communication between the participant and DSN. The app facilitates scheduling sessions, completing CBT exercises between sessions, diabetes self-care documentation, psychometric questionnaires and sending STEADY toolkit documents. Participants will have the option to receive all materials in a paper format or via email instead of the app

14645491, 2024, 4, Downloaded from https://onlinelibrary.wiley.com/doi/10.1111/dme.15273 by <Shibboleth>-member@kcl.ac.uk, Wiley Online Library on [18/10/2024]. See the Terms and Conditions (https://onlinelibrary.wiley.com/terms-and-conditions) on Wiley Online Library for rules of use; OA articles are governed by the applicable Creative Commons License

if they prefer. The STEADY app and digital health platform are hosted by Living With (www.livingwith.health). A study smartphone will be provided for participants who do not have access to a smartphone who wish to use the STEADY app. The process of developing the STEADY app and clinician platform will be described in a future publication.

2.9 | STEADY process evaluation

A process evaluation sub-study will be conducted to assess whether the delivery of the intervention is feasible in practice, explore its implementation and refine the processes and intervention according to feedback from participants and clinicians delivering the therapy (Figure 2). Participants interested in taking part in the process evaluation sub-study will be given a specific information sheet and consent form after randomisation.

All participants in the STEADY intervention group, up to five potential participants who were eligible but declined to take part in the RCT, and the study DSN, clinical psychologist, and psychiatrist will be eligible to take part in the process evaluation. The results of the process evaluation will be taken back to the PPI group to share reflection and plan for the definitive trial. The process of delivery and implementation will be evaluated, and the intervention will be refined.

2.9.1 | Acceptability, appropriateness and feasibility and fidelity of receipt

The Acceptability of Intervention Measure (AIM), Intervention Appropriateness Measure (IAM), and Feasibility of Intervention Measure (FIM) will be used to measure the acceptability, appropriateness and feasibility of STEADY,[21] all three groups in the process evaluation sub-study will complete these measures. To complement these standardised scales, we will conduct semi-structured interviews (approx. 30–60 min) to collect detailed data of the fidelity of specific elements of the intervention. We will include questions exploring participants' experiences in engaging with the therapy, understanding the education and the therapy tools and applying the skills outside of their therapy sessions.

To understand the barriers that may prevent people from taking part in this intervention, we will conduct interviews with up to five participants who were eligible to take part in the STEADY trial but declined. We will collect responses for the AIM, IAM, and FIM, as well as a 30-min semi-structured interview. This will provide more detailed information regarding the barriers to taking part, and improvements that can be made in future iterations of this intervention. After declining to take part, participants will be provided with information about the process evaluation and the importance of understanding barriers to take part and how to improve the intervention for future iterations. Participants will be provided with a £25 shopping voucher as compensation for their time.

To understand barriers and facilitators to the delivery of STEADY and what can be improved, the study DSN, clinical psychologist and psychiatrist supporting STEADY therapy will complete the AIM, IAM and FIM and take part in a 1-h semi-structured interview. This will be completed when all participants in the STEADY intervention group have completed all sessions of STEADY therapy.

2.9.2 | Fidelity of delivery

To assess if the intervention was delivered as intended, the therapy sessions in the STEADY intervention group will be audio-recorded and evaluated according to the Revised Cognitive Therapy Scale (CTS-R), a scale for assessing therapist competence in cognitive therapy.[22] In addition to signing a consent form to the sub-study, the STEADY DSN will ask for the participant's consent to record before each session. As the sub-study is optional, it is not mandatory for participants to record sessions, and they may request the recording, or their participation in the sub-study to be stopped at any time without their care or participation in the main trial being affected.

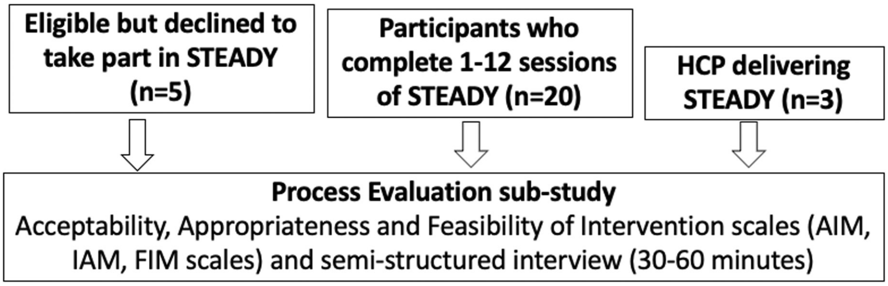

FIGURE 2 Process evaluation sub-study flow chart.

14645491, 2024, 4, Downloaded from https://onlinelibrary.wiley.com/doi/10.1111/dme.15273 by <Shibboleth>-member@kcl.ac.uk, Wiley Online Library on [18/10/2024]. See the Terms and Conditions (https://onlinelibrary.wiley.com/terms-and-conditions) on Wiley Online Library for rules of use; OA articles are governed by the applicable Creative Commons License

3 | PLANNED ANALYSIS

3.1 | Outcomes

The main outcome is the feasibility of the intervention delivery, dropout rates and participant satisfaction. The main biomedical outcome is glycaemia [measured by HbA1c and glucose time in range (TIR)] in the intervention vs. control group at baseline and study end.

Secondary outcomes include proof-of-concept that this intervention improves diabetes management outcomes (HbA1c, TIR, reduction of annualised DKA and severe hypoglycaemia rates, and hospital admissions), mental health outcomes assessed by PROMs, measuring the time needed to collect and analyse data, fidelity of the intervention delivery, and feasibility of introducing a mobile phone app to facilitate intervention delivery. Completion of the STEADY intervention will be defined as attendance of at least 6 sessions.

In a future definitive RCT, we propose HbA1c and TIR in the intervention vs control group at baseline and study end as the primary outcome, and mental health outcomes assessed by PROMs and change in diabetes management outcomes (HbA1c, TIR, reduction of annualised DKA and severe hypoglycaemia rates and hospital admissions as secondary outcomes.

3.2 | Person-centred outcomes

The STEADY PPI group highlighted the importance of measuring recovery through experiential person-centred outcomes of behaviour and quality of life. The PPI group recommended measuring the presence of intrusive thoughts, emotional reactions and behavioural reactions as essential outcomes. To do this, we included 11 PROMs that focus on mood, quality of life and diabetes or eating-disordered health behaviours (Table 2).

3.3 | Data collection

We created an electronic case report form (eCRF) using REDCap, which is GDPR and HIPPA compliant.[23] All data are anonymised and stored using a unique participant identifier. Data will be entered into REDCap directly, including questionnaires and relevant source data such as CGM and glucose meter downloads.

3.4 | Statistical evaluation

Recruitment and dropout rates will be calculated and presented with 95% confidence intervals (CI). The pooled standard deviation (SD) of HbA1c will be calculated, as well as TIR at baseline and study end. The change in HbA1c and TIR will be calculated for both intervention and control groups, and descriptive statistics will be presented to provide information about proof-of-concept for the effectiveness of STEADY; however, these results will not be compared using a statistical test since this feasibility study is not powered for such a comparison.

3.5 | Sample size calculation

A sample size of 40 participants provides sufficient precision for the estimation of a pooled SD for HbA1c, for subsequent use in a sample size calculation of a definitive trial. Assuming an SD of 2% (based on the eligible range of HbA1c of up to 15%), this gives a 95% CI of 1.64% to 2.57%.

3.6 | Precision of the attrition rate

Assuming an attrition rate of 10%, our sample size gives a 95% CI of 3% to 24%. This will provide sufficient precision for the calculation of rates such as recruitment rates. All calculations were performed using the PASS v15 software.

An intention-to-treat as well as per-protocol analysis will be conducted, comparing the difference in outcomes at follow-up against the baseline.

4 | DISSEMINATION

We will report the results of this trial to the study participants, the PPI group, and stakeholders through written and verbal reports. We will present the findings of this trial at relevant national and international conferences and in peer-reviewed scientific journals.

ACKNOWLEDGEMENTS

We would like to thank all people with lived experience and healthcare professionals who attended the STEADY Experience Based Co-Design workshops for their contributions to the STEADY toolkit. In addition, we would like to thank the STEADY PPI group members for their input and support in the development of the STEADY intervention protocol.

FUNDING INFORMATION

This work was conducted as part of the National Institute for Health Research (NIHR) funded STEADY project (Safe management of people with Type 1 diabetes and EAting

14645491, 2024, 4, Downloaded from https://onlinelibrary.wiley.com/doi/10.1111/dme.15273 by <Shibboleth>-member@kcl.ac.uk, Wiley Online Library on [18/10/2024]. See the Terms and Conditions (https://onlinelibrary.wiley.com/terms-and-conditions) on Wiley Online Library for rules of use; OA articles are governed by the applicable Creative Commons License

Disorders studY; CS-2017-17-023) which aims to develop a complex intervention for people with disordered eating and type 1 diabetes N.Z.'s salary was funded by NIHR via the NIHR Clinician Scientist award to MS; J. T. and K. I. are partly funded by the NIHR Mental Health Biomedical Research Centre at South London and Maudsley NHS Foundation Trust and King's College London. M.S. was funded through the National Institute of Health Research (NIHR) NIHR Clinician Scientist Fellowship and Academy of Medical Sciences Starter Grant for Clinical Lecturers (2017); AH's and JB's salaries were in part funded by the NIHR Clinician Scientist Award (CS-2017- 17-023) to MS. The views expressed are those of the author(s) and not necessarily those of the NHS, the NIHR or the Department of Health. AH is supported by the Medical Research Council.

CONFLICT OF INTEREST STATEMENT

None to declare.

ORCID

Natalie Zaremba https://orcid.org/0000-0002-1720-1621
Amy Harrison https://orcid.org/0000-0002-3771-9735
David Hopkins https://orcid.org/0000-0002-0451-0900
Marietta Stadler https://orcid.org/0000-0001-6869-9960

REFERENCES

1. Clery P, Stahl D, Ismail K, Treasure J, Kan C. Systematic review and meta-analysis of the efficacy of interventions for people with type 1 diabetes mellitus and disordered eating. *Diabet Med*. 2017;34(12):1667-1675. doi:10.1111/dme.13509
2. Jones JM, Lawson ML, Daneman D, Olmsted MP, Rodin G. Eating disorders in adolescent females with and without type 1 diabetes: cross sectional study. *BMJ*. 2000;320(7249):1563-1566.
3. Colton P, Olmsted M, Daneman D, Rydall A, Rodin G. Disturbed eating behavior and eating disorders in preteen and early teenage girls with type 1 diabetes: a case-controlled study. *Diabetes Care*. 2004;27(7):1654-1659.
4. Colton PA, Olmsted MP, Daneman D, Rydall AC, Rodin GM. Five-year prevalence and persistence of disturbed eating behavior and eating disorders in girls with type 1 diabetes. *Diabetes Care*. 2007;30(11):2861-2862. doi:10.2337/dc07-1057
5. Bachle C, Lange K, Stahl-Pehe A, et al. Symptoms of eating disorders and depression in emerging adults with early-onset, long-duration type 1 diabetes and their association with metabolic control. *PloS ONE*. 2015;10(6):e0131027. doi:10.1371/journal.pone.0131027
6. Bachle C, Stahl-Pehe A, Rosenbauer J. Disordered eating and insulin restriction in youths receiving intensified insulin treatment: results from a nationwide population-based study. *Int J Eat Disord*. 2016;49(2):191-196. doi:10.1002/eat.22463
7. McClintock JM, Blackmore T, Chepulis LM, Fraser S, Paul RG. The psychological profile of youth and young adults with type 1 diabetes in New Zealand. *Pediatr Diabetes*. 2022;23(1):150-156. doi:10.1111/pedi.13289
8. Goebel-Fabbri AE, Fikkan J, Franko DL, Pearson K, Anderson BJ, Weinger K. Insulin restriction and associated morbidity and mortality in women with type 1 diabetes. *Diabetes Care*. 2008;31(3):415-419. doi:10.2337/dc07-2026
9. Allan J. Understanding poor outcomes in women with type 1 diabetes and eating disorders. *J Diabetes Nurs*. 2015;19:99-103.
10. Merwin RM, Dmitrieva NO, Honeycutt LK, et al. Momentary predictors of insulin restriction among adults with type 1 diabetes and eating disorder symptomatology. *Diabetes Care*. 2015;38(11):2025-2032. doi:10.2337/dc15-0753
11. Vallis M, Jones A, Pouwer F. Managing hypoglycemia in diabetes may be more fear management than glucose management: a practical guide for diabetes care providers. *Curr Diabetes Rev*. 2014;10(6):364-370.
12. Zaremba N, Watson A, Kan C, et al. Multidisciplinary healthcare teams' challenges and strategies in supporting people with type 1 diabetes to recover from disordered eating. *Diabet Med*. 2019;37(12):1992-2000. doi:10.1111/dme.14207
13. Zaremba N, Robert G, Allan J, et al. Developing a novel intervention for type 1 diabetes and disordered eating using a participatory action design process: safe management of people with Type 1 diabetes and EAting Disorders studY (STEADY). *Diabet Med*. 2021;39(4):e14749. doi:10.1111/dme.14749
14. Craig P, Dieppe P, Macintyre S, Michie S, Nazareth I, Petticrew M. Developing and evaluating complex interventions: the new Medical Research Council guidance. *BMJ*. 2008;337:a1655. doi:10.1136/bmj.a1655
15. Staite E, Zaremba N, Macdonald P, et al. "Diabulima" through the lens of social media: a qualitative review and analysis of online blogs by people with Type 1 diabetes mellitus and eating disorders. *Diabet Med*. 2018;35(10):1329-1336. doi:10.1111/dme.13700
16. Harrison A, Zaremba N, Brown J, et al. A cognitive behavioural model of the bidirectional relationship between disordered eating and diabetes self care in people with type 1 diabetes mellitus. *Diabet Med*. 2021;38(7):e14578. doi:10.1111/dme.14578
17. First MB, Williams JBW, Karg R, Spitzer RL. *Structured Clinical Interview for DSM-5 Research Version SCID-5-RV*. American Psychiatric Association; 2015.
18. Markowitz JT, Butler DA, Volkening LK, Antisdel JE, Anderson BJ, Laffel LM. Brief screening tool for disordered eating in diabetes: internal consistency and external validity in a contemporary sample of pediatric patients with type 1 diabetes. *Diabetes Care*. 2010;33(3):495-500. doi:10.2337/dc09-1890
19. Kroenke K, Spitzer RL, Williams JB. The PHQ-9: validity of a brief depression severity measure. *J Gen Intern Med*. 2001;16(9):606-613. doi:10.1046/j.1525-1497.2001.016009606.x
20. Spitzer RL, Kroenke K, Williams JB, Löwe B. A brief measure for assessing generalized anxiety disorder: the GAD-7. *Arch Intern Med*. 2006;166(10):1092. doi:10.1001/archinte.166.10.1092
21. Weiner BJ, Lewis CC, Stanick C, et al. Psychometric assessment of three newly developed implementation outcome measures. *Implement Sci*. 2017;12(1):108. doi:10.1186/s13012-017-0635-3
22. Blackburn IM, Bishop S, Glen AI, Whalley LJ, Christie JE. The efficacy of cognitive therapy in depression: a treatment trial using cognitive therapy and pharmacotherapy, each alone and in combination. *Br J Psychiatry*. 1981;139:181-189.
23. Harris PA, Taylor R, Minor B, et al. The REDCap consortium: building an international community of software platform

14645491, 2024, 4, Downloaded from https://onlinelibrary.wiley.com/doi/10.1111/dme.15273 by <Shibboleth>-member@kcl.ac.uk, Wiley Online Library on [18/10/2024]. See the Terms and Conditions (https://onlinelibrary.wiley.com/terms-and-conditions) on Wiley Online Library for rules of use; OA articles are governed by the applicable Creative Commons License

partners. *J Biomed Inform*. 2019;95:103208. doi:10.1016/j.jbi.2019.103208
24. Fisher L, Polonsky WH, Hessler DM, et al. Understanding the sources of diabetes distress in adults with type 1 diabetes. *J Diabetes Complications*. 2015;29(4):572-577. doi:10.1016/j.jdiacomp.2015.01.012
25. Gonder-Frederick LA, Schmidt KM, Vajda KA, et al. Psychometric properties of the hypoglycemia fear survey-ii for adults with type 1 diabetes. *Diabetes Care*. 2011;34(4):801-806. doi:10.2337/dc10-1343
26. Carver CS, White TL. Behavioral inhibition, behavioral activation, and affective responses to impending reward and punishment: the BIS/BAS scales. *J Pers Soc Psychol*. 1994;67(2):319-333. doi:10.1037/0022-3514.67.2.319
27. Gideon N, Hawkes N, Mond J, Saunders R, Tchanturia K, Serpell L. Development and psychometric validation of the EDE-QS, a 12 item short form of the eating disorder examination questionnaire (EDE-Q). *PLoS ONE*. 2016;11(5):e0152744. doi:10.1371/journal.pone.0152744
28. Holmes-Truscott E, Skovlund SE, Hendrieckx C, Pouwer F, Peyrot M, Speight J. Assessing the perceived impact of diabetes on quality of life: psychometric validation of the DAWN2 impact of diabetes profile in the second diabetes MILES—Australia (MILES-2) survey. *Diabetes Res Clin Pract*. 2019;150:253-263. doi:10.1016/j.diabres.2019.03.020
29. Beecham J, Knapp M. Costing psychiatric interventions. In: Thornicroft G, ed. Gaskell; 2001:200-224.
30. The WHOQOL Group. Development of the World Health Organization WHOQOL-BREF quality of life assessment. *Psychol Med*. 1998;28(3):551-558. doi:10.1017/s0033291798006667
31. Bellace DL, Tesser R, Berthod S, et al. The Yale-Brown-Cornell eating disorders scale self-report questionnaire: a new, efficient tool for clinicians and researchers. *Int J Eat Disord*. 2012;45(7):856-860. doi:10.1002/eat.22023

How to cite this article: Zaremba N, Harrison A, Brown J, et al. Protocol for the STEADY intervention for type 1 diabetes and disordered eating: Safe management of people with Type 1 diabetes and EAting Disorders studY. *Diabet Med*. 2024;41:e15273. doi:10.1111/dme.15273

14645491, 2024, 4, Downloaded from https://onlinelibrary.wiley.com/doi/10.1111/dme.15273 by <Shibboleth>-member@kcl.ac.uk, Wiley Online Library on [18/10/2024]. See the Terms and Conditions (https://onlinelibrary.wiley.com/terms-and-conditions) on Wiley Online Library for rules of use; OA articles are governed by the applicable Creative Commons License
