# Supplementary material for: Safety of a co-designed cognitive behavioural therapy intervention for people with type 1 diabetes and eating disorders (STEADY): a feasibility randomised controlled trial
Source: Lancet Reg Health Eur. 2025 Jan 20;50:101205. doi: 10.1016/j.lanepe.2024.101205 (PMC11788855; doi:10.1016/j.lanepe.2024.101205)
Supplement: Supplemental Table S2 [file mmc4.docx]

**Supplemental Table 2. List of Person Reported Outcomes Measures (PROM)**

| **PROM** | **Psychometrics** |
| --- | --- |
| Diabetes Eating Problems Survey Revised (DEPS-R) (14) | Eating disorder behaviours in T1D |
| Patient Health Questionnaire (PHQ-9) (15) | Standardised mood measure |
| Generalised Anxiety Questionnaire (GAD-7) (16) | Standardised anxiety measure |
| Eating Disorder Examination Questionnaire Short (EDE-QS) (13) | Eating disorders examination short form questionnaire used routinely in eating disorder services |
| Hypoglycaemia Fear Survey HFS-II) (31) | Hypoglycaemia fear survey for adults with T1D with behaviour and worry subscales |
| Preoccupations subscale of the Yale-Brown-Cornell Eating Disorder Scale Self-Report Questionnaire (YBC-EDS-SRQ) (32) | Yale-Brown-Cornell Eating Disorder Scale Self-Report Questionnaire measure eating and weight related preoccupations |
| DAWN2 Impact of Diabetes Profile (DIDP) (33) | DAWN impact of diabetes profile, measures impact of T1D on quality of life |
| Behavioural Inhibition/Behavioural Activation Scales (BIS/BAS) (34) | Behavioural inhibition and activation scales, with subscales measuring drive, reward responsiveness, fun seeking, and inhibition. |
| Diabetes Distress Screening Scale for Adults with Type 1 Diabetes (T1-DDS) (35) | Diabetes distress for T1D, with subscales measuring distress relating to feeling of powerlessness, diabetes management, hypoglycaemia, negative social perception, eating, physician, friends and family. |
| World Health Organization Quality of Life Assessment (WHOQOL (36)) | General quality of life measure with subscales measuring physical health, psychological health, social relationships, and environmental health |
