# Supplementary material for: Safety of a co-designed cognitive behavioural therapy intervention for people with type 1 diabetes and eating disorders (STEADY): a feasibility randomised controlled trial
Source: Lancet Reg Health Eur. 2025 Jan 20;50:101205. doi: 10.1016/j.lanepe.2024.101205 (PMC11788855; doi:10.1016/j.lanepe.2024.101205)
Supplement: Supplemental Table S3 [file mmc5.docx]

**Supplementary Table 3. Withdrawals and losses to follow-up by study arm.**

|  | STEADY (N=20) | Control  (N=20) |
| --- | --- | --- |
| Withdrawals | | |
| N |  |  |
| Withdrew from intervention, total n (%) | 4 (20%) |  |
| Prior to 3 sessions of therapy (did not complete intervention) | 0 |  |
| Prior to 6 sessions of therapy (did not complete intervention) | 3 (15%) |  |
| Prior to 12 sessions (completed intervention; 6+ sessions) | 1 (5%) |  |
| Reasons for withdrawal n (%) |  |  |
| No longer willing to participate | 2 (15%) |  |
| Time demands too great | 1 (5%) |  |
| Lost to follow-up | 0 | 2 (10%) |
| Unable to complete intervention, no longer eligible | 1 (5%) |  |
| Data availability of withdrawals |  |  |
| Data for intention to treat analysis unavailable | 0 | 1 |
