# Supplementary material for: Safety of a co-designed cognitive behavioural therapy intervention for people with type 1 diabetes and eating disorders (STEADY): a feasibility randomised controlled trial
Source: Lancet Reg Health Eur. 2025 Jan 20;50:101205. doi: 10.1016/j.lanepe.2024.101205 (PMC11788855; doi:10.1016/j.lanepe.2024.101205)
Supplement: Supplemental Table S4 [file mmc6.docx]

**Supplemental Table 4. Safety outcome data. Adverse events by study arm (n=11)**

|  |  | **STEADY**  **N=20** | **Control**  **N=20** | **Total**  **N=40** |
| --- | --- | --- | --- | --- |
| **Serious Adverse Events** | **Total n, people (events)** | **5 (5)** | **1 (1)** | **6 (6)** |
| Hospital admission – diabetic ketoacidosis | n, people (events) | 1 | 1 (1) | 1 (1) |
| Hospital admission – hyperglycaemia | n, people (events) | 2 (2) | 0 | 2 (2) |
| Hospital admission - severe hypoglycaemia | n, people (events) | 0 | 0 | 0 |
| Hospital admission - Mental Heath | n, people (events) | 1(1) | 0 | 1 (1) |
| Hospital admission – cardiovascular (subarachnoid bleed) | n, people (events) | 1 (1) | 0 | 1 (1) |
| Hospital admission – gastrointestinal (gallstones) | n, people (events) | 1 (1) | 0 | 1 (1) |
|  |  |  |  |  |
| **Adverse Events** | **Total n, people (events)** | **3 (3)** | **2 (2)** | **5 (5)** |
| Workplace injury | n, people (events) | 1 (1) | 0 | 1 (1) |
| Flu, dehydration | n, people (events) | 2 (2) | 0 | 2 (2) |
| Cataracts operation (planned) | n, people (events) | 0 | 1 (1) | 1 (1) |
| Coronary artery bypass surgery (planned) | n, people (events) | 0 | 1 (1) | 1 (1) |
