# Supplementary material for: Safety of a co-designed cognitive behavioural therapy intervention for people with type 1 diabetes and eating disorders (STEADY): a feasibility randomised controlled trial
Source: Lancet Reg Health Eur. 2025 Jan 20;50:101205. doi: 10.1016/j.lanepe.2024.101205 (PMC11788855; doi:10.1016/j.lanepe.2024.101205)
Supplement: Supplemental Table S5 [file mmc7.docx]

**Supplemental Table 5. Diabetes therapy and psychotropics.**

Use of diabetes therapy modality, use of adjunct diabetes medication and use of psychotropic medication. Data are n (%), median (IQR), mean (SD), or n/N (%). MDI= multiple daily insulin injections; CSII= continuous subcutaneous insulin infusion; HCL=hybrid closed loop therapy; CGMS=continuous glucose monitoring system; SGLT-2=sodium-glucose-co-transporter-2; GLP-1= glucagon like peptide-1; SSRI= selective serotonine uptake inhibitors; SNRI=Serotonin and norepinephrine reuptake inhibitors (SNRIs); NASSA=Noradrenergic and specific serotonergic antidepressants; TCA=tricyclical antidepressants.

|  | N / N | STEADY (n=20) | Control (n=20) |
| --- | --- | --- | --- |
| **Diabetes therapy modality** |  |  |  |
| MDI | 20/20 | 13 (65%) | 10 (50%) |
| CSII | 20/20 | 7 (35%) | 10 (50%) |
| Using Sensor Augmented Therapy | 20/20 | 3(15%) | 4 (20%) |
| Using HCL | 13/10 | 2 | 2 |
| **Type of CSII** |  | | |
| Omnipod® | 4/10 | 4 (20%) | 5 (25%) |
| Tandem T Slim® | 0/10 | 0(0%) | 1(5%) |
| Medtronic® | 7/10 | 7 (35%) | 4 (50%) |
| **Modality of Glucose Testing** |  | | |
| Finger Prick | 20/20 | 1(5%) | 0(0%) |
| Flash Glucose Sensor | 20/20 | 15(75%) | 15 (75%) |
| CGMS | 20/20 | 5(25%) | 5 (25%) |
| **Type of Glucose Sensor** |  | | |
| Libre® | 20/20 | 15 (75%) | 17 (85%) |
| Dexcom® | 20/20 | 3 (15%) | 1(5%) |
| Medtronic® | 20/20 | 2 (10%) | 2(10%) |
| **Adjunct Therapy** |  |  |  |
| Metformin | 20/20 | 3(15%) | 2 (10%) |
| SGLT-2 inhibitor | 20/20 | 0(0%) | 2 (105) |
| GLP-1 agonist | 20/20 | 1(5%) | 0(0%) |
|  |  |  |  |
| **On Psychotropic Medications** | 20/20 | 7(35%) | 10(50%) |
| SSRI |  | 3(15%) | 8 (40%) |
| SNRI |  | 3(15%) | 2 (10%) |
| NASSA |  | 3(15%) | 0 (0%) |
| TCA |  | 1(5%) | 0 (0%) |
| Other Antidepressant |  | 2 (10%) | 0 (0%) |
| 1^st^ Generation antipsychotic |  | 0 (0%) | 1(5%) |
| 2^nd^ Generation antipsychotic |  | 1(5%) | 0 (0%) |
| Hypnotics/ Benzodiazepines/ Daizepam/Alzopram/ Zolpidem |  | 3(15%) | 0 (0%) |
