# Supplementary material for: Safety of a co-designed cognitive behavioural therapy intervention for people with type 1 diabetes and eating disorders (STEADY): a feasibility randomised controlled trial
Source: Lancet Reg Health Eur. 2025 Jan 20;50:101205. doi: 10.1016/j.lanepe.2024.101205 (PMC11788855; doi:10.1016/j.lanepe.2024.101205)
Supplement: Supplemental Table S6 [file mmc8.docx]

**Supplemental Table 6. Diabetes technology subgroup analysis.**

**Baseline characteristics of the intention-to-treat population of the STEADY trial with % CGM wear over 28 day period > 70% and paired time in range data.**

Data are n (%), median (IQR), mean (SD), or n/N (%). CGM=continuous subcutaneous glucose monitor; HbA1c= glycated haemoglobin A1c; BMI=body-mass index; TIR= glucose time in range measured with flash- or continuous glucose monitoring; MDI= multiple daily injection therapy; CSII= continuous subcutaneous insulin infusion pump.

|  | N (11/7)  (STEADY/Control) | STEADY (n=.11.) | Control (n=.7) |
| --- | --- | --- | --- |
| Sex |  |  |  |
| Female | 10/7 | 10 (90.9 %) | 7 (100%) |
| Male | 1/0 | 1 (9.1%) | 0 (0%) |
| Age (years) | 11/7 | 39.2 (26.7 – 57.4) | 34.1 (32.6 – 45.1) |
| Ethnic origin |  |  |  |
| White – English/Welsh/Scottish/ Northern Irish/ British | 9/5 | 9 (81.8 %) | 5 (71.4 %) |
| White – Gypsy or Irish Traveller | 1/0 | 1 (9.1%) | 0 (0%) |
| White- any other background | 0/1 | 0 (0%) | 1 (14.3%) |
| Black - Caribbean | 0/1 | 0 (0 %) | 1 (14.3 %) |
| Asian/ British Pakistani | 1/0 | 1 (9.1%) | 0 (0 %) |
| Diabetes duration (years) | 11/7 | 17 (10 – 30) | 26 (9 – 30) |
| CGM Wear (%) (median, IQR) | 11/7 | 95 (85 – 100) | 100 (88 – 100) |
| HbA1c (mmol/mol) |  | Mean (SD) |  |
| HbA1c (%) | 11/7 | 8.8 (2.5) | 11.0 (2.0) |
| BMI (kg/m^2^) | 11/7 | 25.5 (5.9) | 27.4 (3.5) |
| TIR (%) | 11/7 | 30 (25.2) | 47.9 (25.2) |
| Diabetes therapy modality |  |  |  |
| MDI | 6/6 | 6 (54.5 %) | 6 (85.7%) |
| CSII | 5/1 | 5 (45.5%) | 6 (14.3%) |
