# Supplementary material for: Safety of a co-designed cognitive behavioural therapy intervention for people with type 1 diabetes and eating disorders (STEADY): a feasibility randomised controlled trial
Source: Lancet Reg Health Eur. 2025 Jan 20;50:101205. doi: 10.1016/j.lanepe.2024.101205 (PMC11788855; doi:10.1016/j.lanepe.2024.101205)
Supplement: Supplemental Table S7 [file mmc9.docx]

**Supplementary Table 7: Change of diabetes technology during the trial in STEADY and control groups**

Data are n (%) CGM=continuous subcutaneous glucose monitor; MDI= multiple daily injection therapy.

|  | STEADY  N = 16 | Control  N = 18 |
| --- | --- | --- |
| Change from insulin pump without integrated CGM to insulin pump with commercially available closed loop  N (%) | 3 (18%)  (Omnipod with libre to Omnipod 5 with Dexcom; Omnipod with libre to Tandem x2 with Dexcom; Medtronic 780 with libre to Medtronic 780 with guardian and closed loop) | 1 (5.5%)  (Medtronic with libre to Medtronic closed loop) |
| Change from MDI to insulin pump with closed loop  N (%) |  | 1 (5.5%)  (MDI with libre to Omnipod 5 with Dexcom) |
