# Supplementary material for: Safety of a co-designed cognitive behavioural therapy intervention for people with type 1 diabetes and eating disorders (STEADY): a feasibility randomised controlled trial
Source: Lancet Reg Health Eur. 2025 Jan 20;50:101205. doi: 10.1016/j.lanepe.2024.101205 (PMC11788855; doi:10.1016/j.lanepe.2024.101205)
Supplement: Supplemental Table S8 [file mmc10.docx]

**Supplementary Table 8: Previous experience in therapy / referrals during study**

Data are n (%); CBT= cognitive behavourial therapy

|  | N (STEADY / Control) | STEADY | Control |
| --- | --- | --- | --- |
| Previous experience with therapy at baseline | 20 / 20 | 16 (80%) | 15 (75%) |
| Previous experience with eating disorder therapy at baseline | 20 / 20 | 7 (35%) | 8 (40%) |
| Undertaking therapy as part of usual care during study period | 16 / 18 | 0 | 1 (5.5%)  (CBT therapy, self-referred) |
| Referral to mental health services by usual care at study end, but on waiting list | 16 / 18 | 3 (18.8%)  (1 grief counselling, 1 eating disorder services, 1 other psychiatric) | 2 (11.1%)  (2 eating disorder services) |
