# Supplementary material for: Safety of a co-designed cognitive behavioural therapy intervention for people with type 1 diabetes and eating disorders (STEADY): a feasibility randomised controlled trial
Source: Lancet Reg Health Eur. 2025 Jan 20;50:101205. doi: 10.1016/j.lanepe.2024.101205 (PMC11788855; doi:10.1016/j.lanepe.2024.101205)
Supplement: Supplemental Table S9 [file mmc11.docx]

**Supplementary Table 9: Client Service Receipt Inventory at baseline and follow up.**

Total sum and median (interquartile range) number of contacts between participants and diabetes and mental health professional contacts and emergency services at baseline and study end before and during the trial in STEADY and usual care arms. Data are n, median (interquartile range).

|  | Baseline N Total (Steady / Control) | Baseline sum for all participants (STEADY/ Control) | Baseline Total Median (IQR) | Baseline STEADY median (IQR) | Baseline Control median (IQR) | End N Total (Steady / Control) | End sum for all participants (STEADY / Control) | End Total Median (IQR) | End STEADY median (IQR) | End Control median (IQR) |
| --- | --- | --- | --- | --- | --- | --- | --- | --- | --- | --- |
| **General Practice contacts** | | | | | |  | | | | |
| GP | 39 (19/20) | 212 (110 / 102) | 4 (2,6) | 4 (2, 7) | 3 (1.25, 5.75) | 30 (13 / 17) | 156 (61 / 95) | 3 (0, 10) | 3 (1, 9.5) | 3 (0, 10) |
| Practice Nurse | 39 (19/20) | 64 (33 / 31) | 1 (0, 2) | 1 (0, 3) | 1 (1, 2) | 30 (13 / 17) | 149 (18 / 131) | 1.5 (0, 3) | 1 (0, 3) | 2 (1, 3) |
| **Diabetes Specialist contacts** | | | | | |  | | | | |
| Diabetes Specialist Nurse | 39 (19/20) | 179 (81 / 98) | 2 (1, 4) | 3 (1, 4) | 2 (1, 4) | 30 (13 / 17) | 86 (44 / 42) | 2 (1, 3) | 1 (0.5, 6.5) | 2 (1, 3) |
| Diabetes Consultant | 39 (19/20) | 58 (30 / 28) | 1 (1, 2) | 1 (1, 2) | 1 (0.25, 2) | 30 (13 / 17) | 31 (19 / 12) | 1 (0, 1.25) | 1 (1, 2) | 1 (0, 1) |
| **Mental Health Specialist contacts** | | | | | |  | | | | |
| Psychiatrist contacts | 39 (19/20) | 51 (31 / 20) | 0 (0, 0) | 0 (0, 1) | 0 (0, 0) | 30 (13 / 17) | 20 (11 / 9) | 0 (0, 0.25) | 0 (0, 1.5) | 0 (0, 0) |
| Clinical psychologist | 38 (18/20) | 79 (37 / 42) | 0 (0, 0.25) | 0 (0, 2) | 0 (0, 3.75) | 30 (13 / 17) | 57 (18 / 39) | 0 (0, 0.25) | 0 (0, 0) | 0 (0, 1) |
| Other mental health professional | 39 (19/20) | 198 (173 / 25) | 0 (0, 2) | 0 (0, 7) | 0, (0, 0.75) | 30 (13 / 17) | 92 (78 / 14) | 0 (0, 1) | 0 (0, 6) | 0 (0, 0) |
| **Planned visits to hospital** | | | | | |  | | | | |
| Planned visits to hospital – T1D related | 38 (18/20) | 20 (15 / 5) | 0 (0, 1) | 0 (0,1) | 0 (0, 0.75) | 30 (13 / 17) | 5 (2 / 3) | 0 (0, 0) | 0 (0, 0) | 0 (0, 0) |
| Planned visits to hospital – not T1D related | 39 (19/20) | 22 (6 / 16) | 0 (0, 1) | 0 (0, 0) | 0 (0, 1) | 30 (13 / 17) | 21 (12 / 9) | 0 (0, 1) | 0 (0, 2) | 0 (0, 0) |
| **Emergency services contacts** | | | | | |  | | | | |
| A&E attendance - T1D related | 39 (19/20) | 0 (0 / 0) | N/A | N/A | N/A | 30 (13 / 17) | 0 (0/0) | 0 (0, 0) | N/A | N/A |
| Ambulance calls - T1D related | 39 (19/20) | 2 (1 / 1) | 0 (0, 0) | 0 (0,0) | 0 (0,0) | 30 (13 / 17) | 3 (2 / 1) | 0 (0, 0) | 0 (0, 0) | 0 (0, 0) |
| A&E attendance - unrelated to T1D | 39 (19/20) | 14 (4 / 10) | 0 (0, 0) | 0 (0,0) | 0 (0, 0.75) | 30 (13 / 17) | 8 (5 / 3) | 0 (0, 0) | 0 (0, 0) | 0 (0, 0) |
| Ambulance calls - unrelated to T1D | 39 (19/20) | 4 (0 / 4) | 0 (0, 0) | N/A | 0 (0,0) | 30 (13 / 17) | 1 (0 / 1) | 0 (0, 0) | 0 (0, 0) | 0 (0, 0) |
